# Equity and efficiency of public hospitals’ health resource allocation in Guangdong Province, China

**DOI:** 10.1186/s12939-022-01741-1

**Published:** 2022-09-22

**Authors:** Wanmin Su, Liulin Du, Yujun Fan, Peixi Wang

**Affiliations:** 1 School of Nursing and Health, Henan University, Kaifeng, 475004 Henan People’s Republic of China; 2 The Seventh Affiliated Hospital, Southern Medical University, Foshan, Guangdong 528244 People’s Republic of China

**Keywords:** Public hospitals, Health resource allocation, Equity, Efficiency, Guangdong Province-China

## Abstract

**Background:**

To better meet people’s growing demand for medical and health services, 21 cities in Guangdong Province were involved in the reform of public hospitals in 2017. This paper evaluates the equity and efficiency of public hospitals’ health resource allocation in Guangdong Province and explores ways to change the current situation.

**Methods:**

Data were collected from the Guangdong Health Statistical Yearbook 2016–2020 and Guangdong Statistical Yearbook 2017–2021. The Gini coefficient (*G*), Theil index (*T*), and health resource density index (HRDI) were used to measure the equity of health resource allocation. An improved three-stage DEA method was applied in efficiency evaluation. The entropy weight method was employed to calculate the weight of different indicators to obtain a comprehensive indicator representing the overall volume of health resources in each city. A two-dimensional matrix was drawn between the HRDI of the comprehensive indicator and efficiency and the per capita government financial subsidies and efficiency to observe the coordination of equity and efficiency across regions.

**Results:**

From 2016 to 2020, the *G* of public hospital, bed, and health technician allocation by population remained below 0.2, while that by geographical area ranged from 0.4 to 0.6; the *G* of government financial subsidies by population was above 0.4, while that by geographical area was greater than 0.7. The results for *T* showed that inequality mainly comes from intraregional differences, and the Pearl River Delta contributes most to the overall differences. Although the HRDI of the Pearl River Delta is far greater than that of other regions, obvious differences exist across cities in the region. Only 38.1% of cities were found to be efficient in 2020. The Pearl River Delta was in the first quadrant, and the other three regions were in the third quadrant, accounting for a large proportion.

**Conclusion:**

The equity of government financial subsidies allocation was the worst, and there were distinct regional differences in the geographical distribution of health resources. The development of healthcare within the Pearl River Delta was highly unbalanced. The development of healthcare was uneven between the Pearl River Delta, eastern, western, and mountainous regions. In addition, most cities in the eastern, western, and mountainous regions bore the dual pressures of relatively insufficient health resources and inefficiency.

**Supplementary Information:**

The online version contains supplementary material available at 10.1186/s12939-022-01741-1.

## Background

As the main body of China’s medical service system, public hospitals serve as the main force in the implementation of health strategies in China. During the past two years of prevention and control of the COVID-19 pandemic, the percentage of patients treated in public hospitals nationwide exceeded 98%, undertaking the most urgent, difficult, and dangerous tasks and demonstrating the essential role of public hospitals in safeguarding people’s lives, health, and social stability [1]. As health resources are the basis for the delivery of health care services, the well-balanced configuration and efficient utilization of public hospitals’ health resources is a prerequisite for the improvement in health output and services in China. The development of health care in China over the past 70 years also shows that unity of equity and efficiency is an essential requirement for the sustainable development of health services [2, 3]. The evolution of medical and health services in China can be divided into three stages. In the first stage, from the establishment of the People’s Republic of China to the introduction of reform and opening up (1949–1978), the government shouldered the greatest responsibility for healthcare to ensure accessible and affordable healthcare for residents in urban and rural areas in the context of the planned economy management model [4]. During this period, the state invested no more than 3% of its GDP in the health field, and yet it was able to meet the basic health care needs of the entire population [2, 5]. Nevertheless, patterns of administration in the planned economic system discouraged the active participation of medical institutions and staff. Common problems such as poor service quality, low operational efficiency, and intense financial pressure imposed on the government hindered the rapid advancement of health services. The second period was from 1979 to 2009. Following the introduction of the market economy, the government reduced its financial investment in hospitals and increased the autonomy of public hospitals so they could operate on their own [2, 4]. Over this period, the medical service quality and operational efficiency of public hospitals improved as a result of increased market involvement. However, due to deficient input from the government and stimulated by policies, hospitals pursued excessive profitability, causing a rapid rise in medical expenses as well as the emergence of the problem of it being “expensive and difficult to see a doctor”. Although progress was made in some areas, the problems exposed were even more serious; on the whole, the health care reform was not successful [6]. Then, a new round of reform was introduced in 2009, which specified the public welfare aspect of healthcare and highlighted the unified pursuit of equity and efficiency. Concretely, the reform aimed to strengthen the government’s accountability for providing public sanitation and basic medical services in the first place and focus on the role of the market mechanism in the second place [7]. Public hospital reform was the core of this round of medical system reform, and China started pilot projects of public hospital reform in 2010 [4]. As one of the first pilot provinces, Guangdong Province, in accordance with national requirements, conducted trials of public hospital reform in Shenzhen (SZ), Zhuhai (ZH), and Dongguan (DG) [8]. As of 2017, public hospitals throughout 21 cities of Guangdong Province had been involved in the reform [9].

Guangdong Province (20°09′ ~ 25°31′ N, 109°45′ ~ 117°20′ E), located in the southernmost part of mainland China, covers a land area of 179,700 km^2^. Guangdong Province is the largest economy in China and has the largest population; the GDP of Guangdong Province in 2021 was 12.44 trillion yuan, ranking first in the country for 33 consecutive years; the permanent population was 126.84 million, accounting for 8.98% of the country [10, 11]. The key health indicators in the province were at the forefront of the country in 2020, with an average life expectancy of 78.4 years, a maternal mortality rate and infant mortality rate of 10.18 per 100,000 and 2.13 per 1000, respectively [12]. How to utilize the fruits of economic development to satisfy the ever-increasing demands for better healthcare and to complete the crucial mission of constructing a healthy and powerful province has been a great challenge for Guangdong Province.

With the development of the social economy and the popularization of prevention-oriented health concepts, society has paid more attention to equity and efficiency in the health field than ever before, and increasing numbers of studies have explored the equity and efficiency of health resource allocation. The majority of studies have examined the equality and efficiency of the distribution of health resources in all medical and health institutions [13–17], without discriminating between different types of health resources in varied healthcare institutions [18–22]. Few studies have focused on health resources in public hospitals. With the introduction of policies to encourage and guide social capital into the health care field, investment in the medical sector has been increasing [23]. Although the number of private hospitals surpassed that of public hospitals in 2015 for the first time and has remained so since then [24–26], there are substantial discrepancies in resource aggregation and operational efficiency between private and public hospitals [27]. In addition, grassroots healthcare institutions have indeed improved their hardware and equipment, personnel, and service capacity since the start of the new healthcare reform, but the difference has not been narrowed in comparison to public hospitals. All these facts suggest that it is necessary to differentiate the types of health resources in different medical institutions when measuring the equity and efficiency of health resource allocation for more precise conclusions. Moreover, for the most part, studies have analyzed the equity and efficiency of health resource distribution in parallel, neglecting to incorporate equity and efficiency into the same analytical framework.

Different approaches based on data envelopment analysis (DEA) have been widely used as tools to assess the efficiency of decision-making units (DMUs) in the health care sector in China and abroad. The use of these methods can be broadly categorized into three types. The first is the use of a traditional DEA model or an extended model based on DEA to measure the efficiency score of DMUs. The efficiency of several hospital health centers in Greece was investigated with an input-oriented CCR-DEA model, and excellent performance was found for the units additionally providing preventive medical services [28]. By means of the BCC-DEA model, the efficiency of intensive care units in Iran was estimated, and five hospitals were identified as efficient in technical, managerial and scale performance [29]. An output-oriented DEA model was employed to assess the efficiency of maternal and child health resource allocation in Hunan Province, China, and over 40% of regions were found to have poor performance [30]. Chitnis A and Mishra DK used the output-oriented CCR-DEA model and superefficiency DEA model to assess the performance efficiency of 25 Indian private hospitals and determined that the low use of resources was the main reason for the underperformance of hospitals [31]. Second, studies apply the two-stage DEA method, which combines the DEA method with the Tobit regression method, aiming at efficiency evaluation and exploration of influencing factors. An input-oriented DEA model was applied to calculate the efficiency values of 11 Palestinian public hospitals, after which the Tobit regression model was constructed to explore contextual factors. Efficiency scores increased by 4% over the period from 2010 to 2015, and the outpatient-inpatient ratio had a positive effect on hospitals’ efficiency [32]. The efficiency of public and private hospitals in Beijing was analyzed with an output-oriented DEA method, and the findings revealed that the scale efficiency of public hospitals in Beijing was higher than that of private hospitals from 2012 to 2017. Then, the panel Tobit regression implied that some hospital characteristics, such as service type, level and governance structure, influenced the efficiency of public hospitals, while the geographical location impacted the efficiency of private hospitals [33]. A two-stage DEA approach was adopted to examine the technical efficiency of healthcare systems in a sample of 49 lower-middle-income countries and concluded that GDP per capita, percentage of total government health expenditure, and population density probably made a positive contribution to health efficiency [34]. Third, the three-stage DEA approach is used, integrating the conventional DEA model with stochastic frontier analysis (SFA) to adjust for the effects of environmental variables and statistical noise on efficiency values. Resource utilization efficiency in obstetrics and gynecology units was studied with a three-stage DEA method, and the technical efficiency and scale efficiency values of units were 0.48 and 0.54, respectively [35]. By using the three-stage DEA method, the technical efficiency of Chinese regional public hospitals was measured, and the average efficiency was found to improve from 0.927 to 0.981 during the period 2011–2018 [36]. Li et al. integrated input-oriented DEA and SFA to estimate the operational efficiency of basic pension insurance and obtained values of 0.742, 0.689, and 0.505 for the eastern, central and western regions, respectively. Additionally, they found that GDP, urbanization rate, and the amount of government public expenditure positively affected operational efficiency, while the elderly dependency ratio had a significant negative impact on efficiency [37]. In summary, the first approach has no bias adjustment for efficiency scores; the second method is unable to account for the effect of statistical noise on efficiency values, despite its ability to explore the factors influencing efficiency values [38]. For the third method, the three-stage DEA method, random error and disturbance of environmental factors can be effectively removed to put all the DMUs in a homogeneous environment for comparison [38]. However, this method still has some drawbacks. For example, the model used in the first and third stages, which are conventional DEA methods, cannot solve the relaxation problem and sequencing problem of DMUs, which can be easily overcome through the use of the slack-based measure of the superefficiency DEA model (superefficient SBM model), an improved DEA model [39]. Therefore, future research should endeavor to capitalize on the advantages of these two models while performing efficiency analysis.

Concerning the selection of indicators, appropriate inputs and outputs are critical for a meaningful analysis [40]. Most researchers selected input indicators of hospital performance assessment in terms of labor and capital investment [13, 28–30, 33, 36, 41–49]. The number of healthcare institutions and beds were commonly chosen to represent capital investment [33, 42, 46–49]. Regarding labor variables, health workers [13, 17, 42, 44–47, 50], health technical workers [30, 33, 36, 42, 45, 48, 49], physicians [28–30, 36, 41, 43–45, 50], and nurses [17, 28, 30, 36, 41, 42, 44, 45] were often regarded as types of labor investment. In the selection of output indicators, there are two main categories of expected and undesirable outputs. For data availability issues, most studies opt for expected indicators when conducting analysis. The number of outpatient visits or outpatient and emergency visits [13, 28, 30, 36, 41, 42, 44–49, 51], inpatient visits or discharged visits [28, 29, 36, 41–44, 46–48, 51] and revenue [17, 33] are usually regarded as expected outputs, and infection incidence or infectious patients and patient or population mortality [36, 50, 52] are regarded as undesirable outputs. Kohl S and Schoenfelder J et al. pointed out that it is of great importance to distinguish between absolute and relative data to prevent biased conclusions [40]. However, this has been ignored in some studies.

Based on a review and summary of the relevant literature, this study assesses the equality and efficiency of the distribution of public hospitals’ health resources in Guangdong Province with the use of the latest data, aiming to provide a thorough understanding of the postreform development of the health sector, analyze the problems that may exist, and offer appropriate countermeasures for the sustainable development of health services. In addition, this study attempts to make improvements in the following aspects. First, it puts equity and efficiency into one analytical framework following an independent analysis of efficiency and equity. Specifically, all the indicators used for equity analysis are given different weights through the entropy weight method to derive comprehensive indicators representing the total amount of health resources. We then calculate the HRDI of the comprehensive indicator reflecting the volume of health resources in each city in terms of geography and population. Next, two-dimensional matrix diagrams of the HRDI of the comprehensive indicator and efficiency, per capita government subsidies and efficiency are drawn to observe the coordination of the equity and efficiency in health resource allocation across regions. Second, for efficiency analysis, the study constructs an improved three-stage DEA method, which combines the traditional DEA method, SFA and the superefficient SBM model. For this approach, not only the effects of environmental factors and statistical noise on the efficiency but also the ranking of the effective units and slack movement are considered. Third, all data used for efficiency assessment are unified as absolute data, without confusion between absolute and relative values, to prevent biased outcomes.

## Methods

### Data resources, regional division, and statistical analysis

**Fig. 1:**
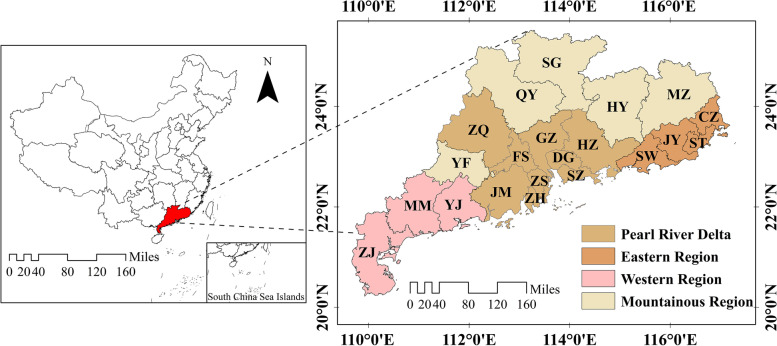
Spatial range and division of the study region

Data for the study were derived from official publications, including Guangdong Health Statistical Yearbook (2016—2020) and Guangdong Statistical Yearbook (2017—2021). A series of longitudinal data were used to evaluate the equitability and efficiency of public hospitals’ health resource allocation in Guangdong Province from 2016 to 2020. Based on the economic development level and geographical position [53], the 21 cities in Guangdong Province were divided into four groups: the Pearl River Delta, eastern region, western region, and mountainous region (detailed information can be seen in Fig. 1). Microsoft Excel 2019 was used to calculate the Gini coefficient as well as the Theil index and draw the Lorenz curve. ArcGIS 10.8 was employed for mapping the distribution of health resources, Frontier 4.1 for SFA analysis, and MaxDEA 8 Ultra for data envelopment analysis.

### Indicators of equity and efficiency

Consistent with most previous studies, this study selects input variables in terms of labor and capital. Three indicators are deemed to be input variables: the first input is the number of health technicians (including doctors, nurses, pharmacists, and other technical personnel) as labor investment; the other two are the numbers of public hospitals and beds as a proxy for capital. The total number of visits (including outpatient, emergency, single health check-up, and health consultation guidance visits), number of hospital discharges, number of health examinations, and annual medical revenue are regarded as output variables. Generally, the number of DMUs is required to be twice the sum of the input variables and output variables to obtain the discriminative power of DEA [40], which is met in this study (21 > 2 × (3 + 4)). A total of four indicators are used in equity analysis: the number of health technicians, number of public hospitals, number of beds, and government financial subsidies to public hospitals.

### Environmental variables

Hospital efficiency can be influenced by managerial inefficiency and environmental factors. Yi M et al. identified five influential factors in terms of economic development, population, and education [14]. On the basis of the aforementioned approach, this study considers the following environmental variables for public hospitals in Guangdong Province with respect to economic development, population, education, insurance, and policy: per capita GDP, urbanization rate, population density, the proportion of high school graduates to the overall permanent population, the proportion of the permanent population covered by basic medical insurance, and the proportion of government health expenditure to financial expenditure.

### Gini coefficients and Lorenz curves

Gini coefficients and Lorenz curves are widely used to assess the equality of health resource allocation [17, 30, 42, 54]. Regarding the Lorenz curves, the x-axis and the y-axis represent the cumulative percentage of the population or geographic area and the cumulative percentage of health resources (hospitals, beds, health technicians, and government financial subsidies), respectively. *G* is equal to the ratio of the area enclosed by the absolute equity line and the Lorenz curve to the area of the right triangle under the absolute equity line. Formula (1) is adopted to compute *G*.

1 $$G=\sum_{i=1}^n{P}_i{Y}_i+2\sum_{i=1}^n{P}_i\times \left(1-{V}_i\right)-1$$

where *P*_*i*_ denotes the proportion of the resident population or geographical area in the *ith* city to the total population or geographical area in the province at the end of the year, *Y*_*i*_ is the proportion of the number of health resources (public hospitals, beds, health technical workers and government financial subsidies) in the *ith* city to the total number of health resources, and *V*_*i*_ is the cumulative percentage of *Y*_*i*_ from *i* = 1 to *i* after ranking the per capita or per square kilometer health resources from the lowest number to the highest number. *G* ranges from 0 to 1. Generally, *G* below 0.2 is absolutely fair; 0.2 to 0.3, fair; >0.3 to 0.4, relatively fair; >0.4 to 0.5, relatively unfair; and above 0.5, very unfair [42, 54].

### Theil index

The Theil index (*T*) is employed to explain the sources of inequality [17, 42]. *T* is between 0 and 1*.* Generally, the closer *T* is to 0, the better the fairness, and vice versa [42, 55]. Formula (2) is adopted to calculate *T*, the value of the total Theil index.

2 $$\begin{array}{c}T=\sum\limits_{i=1}^nP_i\log\frac{P_i}{Y_i}\end{array}$$

where *P*_*i*_ represents the percentage of the population of each city in Guangdong Province and *Y*_*i*_ the percentage of health resources of each city in Guangdong Province. *T* can be decomposed into *T*_*intra*_ and *T*_*inter*_ [22, 42]. The formulas are given as follows:

3 $${\displaystyle \begin{array}{c}T={T}_{intra}+{T}_{inter}\end{array}}$$

4 $$\begin{array}{c}T_{intra}=\sum\limits_{g=1}^kP_gT_g\end{array}$$

5 $${\displaystyle \begin{array}{c}{T}_{inter}=\sum\limits_{g=1}^k{P}_g\log \frac{P_g}{Y_g}\end{array}}$$

where *T*_*g*_ is the Theil index of the four regional groups, including the Pearl River Delta, eastern region, western region, and mountainous region of Guangdong Province. *P*_*g*_ is the proportion of the population of the four regions to the total population of Guangdong Province, and *Y*_*g*_ is the proportion of the health resources of the four regions to the total population of Guangdong Province. *T*_*intra*_ denotes the intraregional distribution of health resources in the four regions. *T*_*inter*_ represents the interregional distribution of health resources among the four regions. The contribution rates of intraregion, interregion, and different regions can be obtained by the following formulas [55, 56].

6 $${\displaystyle \begin{array}{c}\mathrm{contribution}\ \mathrm{rates}\ \mathrm{of}\ \mathrm{intraregion}={T}_{intra}/T\end{array}}$$

7 $${\displaystyle \begin{array}{c}\mathrm{contribution}\ \mathrm{rates}\ \mathrm{of}\ \mathrm{interregion}={T}_{inter}/T\end{array}}$$

8 $${\displaystyle \begin{array}{c}\mathrm{contribution}\ \mathrm{rates}\ \mathrm{of}\ \mathrm{the}\ \mathrm{different}\ \mathrm{regions}={P}_g{T}_g/T\end{array}}$$

### Health Resources Density Index (HRDI)

The HRDI demonstrates the impact of demographic and geographic factors on the concentration of health resources to prevent bias caused by the use of a single population or geographic dimension [17]. The value of HRDI equals the geometric mean of health resources per thousand persons and health resources per square kilometer [22].

### Entropy weight method

The entropy weight method is based on the concept of entropy in thermodynamics, which was first applied to solve problems of information theory by Shannon [57]. It is an objective weighting method based on the dispersion degree of the evaluation index data [58–60]. Basically, the greater the value of entropy is, the more balanced the structure of the system, the smaller the difference coefficient, and the smaller the weight of the indicator [58–60]. This means that the greater the fluctuation of the evaluation index between different evaluation units is, the smaller the entropy value and the larger the weight assigned. In this case, this approach is used to assign weights to the indicators used for equity analysis. Assume that there are m objects for evaluation (*A*_1_, *A*_2_, …, *A*_*m*_) and each has n criteria (*C*_1_, *C*_2_, …, *C*_*n*_), which form the initial decision matrix as follows.

9 $${\displaystyle \begin{array}{c}A=\left[\begin{array}{ccc}{a}_{11}& \cdots & {a}_{1n}\\ {}\vdots & \ddots & \vdots \\ {}{a}_{m1}& \cdots & {a}_{mn}\end{array}\right]={\left[{a}_{ij}\right]}_{m\times n},\left(i=1,2,\cdots, m;j=1,2,\cdots, n\right)\end{array}}$$

The specific calculation steps are presented as follows [61–63].

- Step 1: Dimensionless processing is performed on [*a*_*ij*_]_*m* × *n*_ to eliminate the influence of the index dimension, and the dimensionless data matrix ${\left[{a}_{ij}^{\ast}\right]}_{m\times n}$ is obtained. It can be calculated from:

10 $${\displaystyle \begin{array}{c}{a}_{ij}^{\ast }=\frac{a_{ij}-\min {a}_j}{\max {a}_j-\min {a}_j}\end{array}}$$

- Step 2: Calculate the entropy of the indexes using:

11 $${\displaystyle \begin{array}{c}{e}_j=-\frac{\sum_{i=1}^m{p}_{ij}\ln {p}_{ij}}{\ln m}\end{array}}$$

where

12 $${\displaystyle \begin{array}{c}{p}_{ij}=\frac{a_{ij}^{\ast }}{\sum_{i=1}^m{a}_{ij}^{\ast }}\end{array}}$$

When *p*_*ij*_ = 0, $\underset{p_{ij}\to 0}{\lim }{p}_{ij}\ln {p}_{ij}=0$ is defined.

- Step 3: Compute the entropy weight of each index using:

13 $${\displaystyle \begin{array}{c}{w}_j=\frac{1-{e}_j}{n-\sum_{j=1}^n{e}_j},\sum\limits_{j=1}^n{w}_j=1\end{array}}$$

- Step 4: Calculate the comprehensive indicator using:

14 $${\displaystyle \begin{array}{c}{C}_i=\sum\limits_{j=1}^n{w}_j{a}_{ij}\end{array}}$$

### The improved three-stage DEA method

The three-stage DEA method proposed by Fried et al. can separate the influence of managerial inefficiency, environmental factors, and statistical noise on efficiency, which facilitates a more precise efficiency value [38]. However, it cannot tackle the problems of sequencing of effective units and relaxation amount, which can be dealt with by a slack-based measure of superefficiency, developed by Tone [39]. Therefore, the improved three-stage DEA method is constructed in the study to take full advantage of the two models.

- Stage 1: Input-oriented conventional DEA and input-oriented SBM model of superefficiency

Since the dependent variables in the SFA model are required to be greater than or equal to 0 [38], as above, slack is calculated by applying the input-oriented conventional DEA model under constant returns to scale using the original input and output data. Then, the SBM model of superefficiency is used to calculate the efficiency score. Accounting for the nondiscretionary nature of the outputs, i.e., the fact that hospital managers have no control over the number of patients they treat, and the small elasticity of demand for the outputs, it is more suitable to choose the input-oriented DEA model [22, 28].

- Stage 2: Effects of the environmental variables

In the second stage, the SFA model is used to decompose the input relaxation as follows:

15 $${\displaystyle \begin{array}{c}{S}_{ij}={f}^i\left({Z}_j;{\beta}^i\right)+{v}_{ij}+{u}_{ij},i=1,2,\cdots, m;j=1,2,\cdots, n\end{array}}$$

where *S*_*ij*_ represents the slack variable of the *i* input of *DMU*_*j*_, obtained from stage 1; *f*(*Z*_*j*_; *β*_*i*_) represents the effect of environmental variables on *S*_*ij*_, among which *Z*_*j*_ and *β*_*i*_ are the environmental variable and the coefficient of the environmental variable, respectively; the composite error term consists of *v*_*ij*_ (${v}_{ij}\ \epsilon\ N\left(0,{\sigma}_v^2\right)$ and *u*_*ij*_ (${u}_{ij}\ \epsilon\ {N}^{+}\left(0,{\sigma}_u^2\right)$), representing the influence of random disturbance and managerial inefficiency on input relaxation. Assuming that the distributions of *v*_*ij*_ and *u*_*ij*_ are independent of each other, $\gamma ={\sigma}_u^2/\left({\sigma}_u^2+{\sigma}_v^2\right)$ is defined. The paremeters ( *β*^*i*^, *σ*^2^, *γ*) are estimated by maximum likelihood techniques. Because input slacks require the composite error of the cost function *ε*_*ij*_ = *v*_*ij*_ + *u*_*ij*_, managerial inefficiency is obtained according to Luo as follows [64]:

16 $${\displaystyle \begin{array}{c}E\left({u}_{ij}|{v}_{ij}+{u}_{ij}\right)=\frac{\lambda \sigma}{1+{\lambda}^2}\left[\frac{\phi \left({\varepsilon}_{ij}\lambda /\sigma \right)}{\Phi \left({\varepsilon}_{ij}\lambda /\sigma \right)}+\frac{\varepsilon_{ij}\lambda }{\sigma}\right]\end{array}}$$

where $\sigma =\sqrt{\sigma_u^2+{\sigma}_v^2}$ and *λ* = *σ*_*u*_/*σ*_*v*_; *ϕ*(∙) and Φ(∙) are the density function and distribution function of the standard normal distribution, respectively.

DMUs’ adjusted inputs are calculated by means of following formula:

17 $${\displaystyle \begin{array}{c}{X}_{ij}^A={X}_{ij}+\left[\mathit{\max}\left(f\left({Z}_j;{\hat{\beta}}_i\right)-f\left({Z}_j;{\hat{\beta}}_i\right)\right)\right]+\left[\mathit{\max}\left({v}_{ij}\right)-{v}_{ij}\right],i=1,2,\cdots, m;j=1,2,\cdots, n\end{array}}$$

where *X*_*ij*_ and ${X}_{ij}^A$ represent the observed and adjusted input quantities, respectively. $\left[\mathit{\max}\left(f\left({Z}_j;{\hat{\beta}}_i\right)-\left({Z}_j;{\hat{\beta}}_i\right)\right)\right]$ adjusts all the DMUs into a common operating environment; [*max*(*v*_*ij*_) − *v*_*ij*_] makes all the DMUs face the same statistical noise. Therefore, the input of each DMU is adjusted to the same external environmental conditions and statistical noise.

- Stage 3: Calculation of the efficiency scores

In the third stage, the original input data are substituted with the adjusted input data from (17), keeping the outputs constant. The efficiency of DMUs is evaluated by the input-oriented SBM model of superefficiency under constant returns to scale, as developed by Tone [39]. The superefficiency of (*x*_*o*_, *y*_*o*_) is defined as the optimal objective function value ${\delta}_I^{\ast }$. The efficiency scores for each DMU can be calculated by using the equation below:

18 $${\displaystyle \begin{array}{c}{\delta}_I^{\ast }= min\delta =\frac{1}{m}{\sum}_{i=1}^m\frac{{\overline{x}}_i}{x_{io}},\end{array}}$$

subject to

19 $${\displaystyle \begin{array}{c}\overline{x}\ge \sum\limits_{j=1,j\ne 0}^n{x}_j{\lambda}_j,\end{array}}$$

20 $${\displaystyle \begin{array}{c}\overline{y}\le \sum\limits_{j=1,j\ne 0}^n{y}_j{\lambda}_j,\end{array}}$$

21 $${\displaystyle \begin{array}{c}\ \overline{x}\ge {x}_o,\overline{y}={y}_o,\lambda \ge 0.\end{array}}$$

where ${\delta}_I^{\ast }$ represents SBM-input superefficiency. Suppose there are *n* DMUs, and each DMU has *m* inputs and *s* outputs. The inputs are expressed as *x*_*ij*_ and outputs as *y*_*ij*_. *λ* is a nonnegative vector. DMUs with efficiency scores greater than or equal to 1 indicate efficiency, and higher values indicate more efficiency [39].

## Results

### Current situation of public hospitals’ health resource allocation in Guangdong Province

The data in Table 1 indicate that, except for a slight decrease in the number of public hospitals, the amount of beds, health technicians, and government financial subsidies all increased from 2016 to 2020. Beds, health technicians, and government financial subsidies per thousand persons and per square kilometer also exhibited a growth trend from 2016 to 2020, among which government financial subsidies grew the fastest (Table 1). The numbers of public hospitals, beds, health technicians, and government financial subsidies in the Pearl River Delta accounted for 55.79, 62.85, 69.19, and 74.26%, respectively (detailed information can be seen in Table S1).

**Table 1:** Public hospitals’ health resource allocation in Guangdong Province from 2016 to 2020

Year	Public hospitals			Beds			Health technicians			Government financial subsidies (million)		
	/1000 persons	/km^2^	Total	/1000 persons	/km^2^	Total	/1000 persons	/km^2^	Total	/1000 persons	/km^2^	Total
2016	0.0076	0.0051	909	2.7532	1.8242	327,846	3.3433	2.2162	398,118	0.1950	0.1292	23,218
2017	0.0075	0.0050	907	2.8077	1.8967	340,888	3.4573	2.3366	419,752	0.2391	0.1615	29,033
2018	0.0073	0.0050	898	2.8673	1.9700	354,058	3.5578	2.4455	439,316	0.2817	0.1935	34,783
2019	0.0072	0.0050	900	2.9867	2.0764	373,008	3.6818	2.5596	459,825	0.3299	0.2293	41,205
2020	0.0071	0.0050	898	3.0063	2.1126	379,521	3.7705	2.6496	475,989	0.4816	0.3383	60,796

### The equity of public hospitals’ health resource allocation in Guangdong Province

**Fig. 2:**
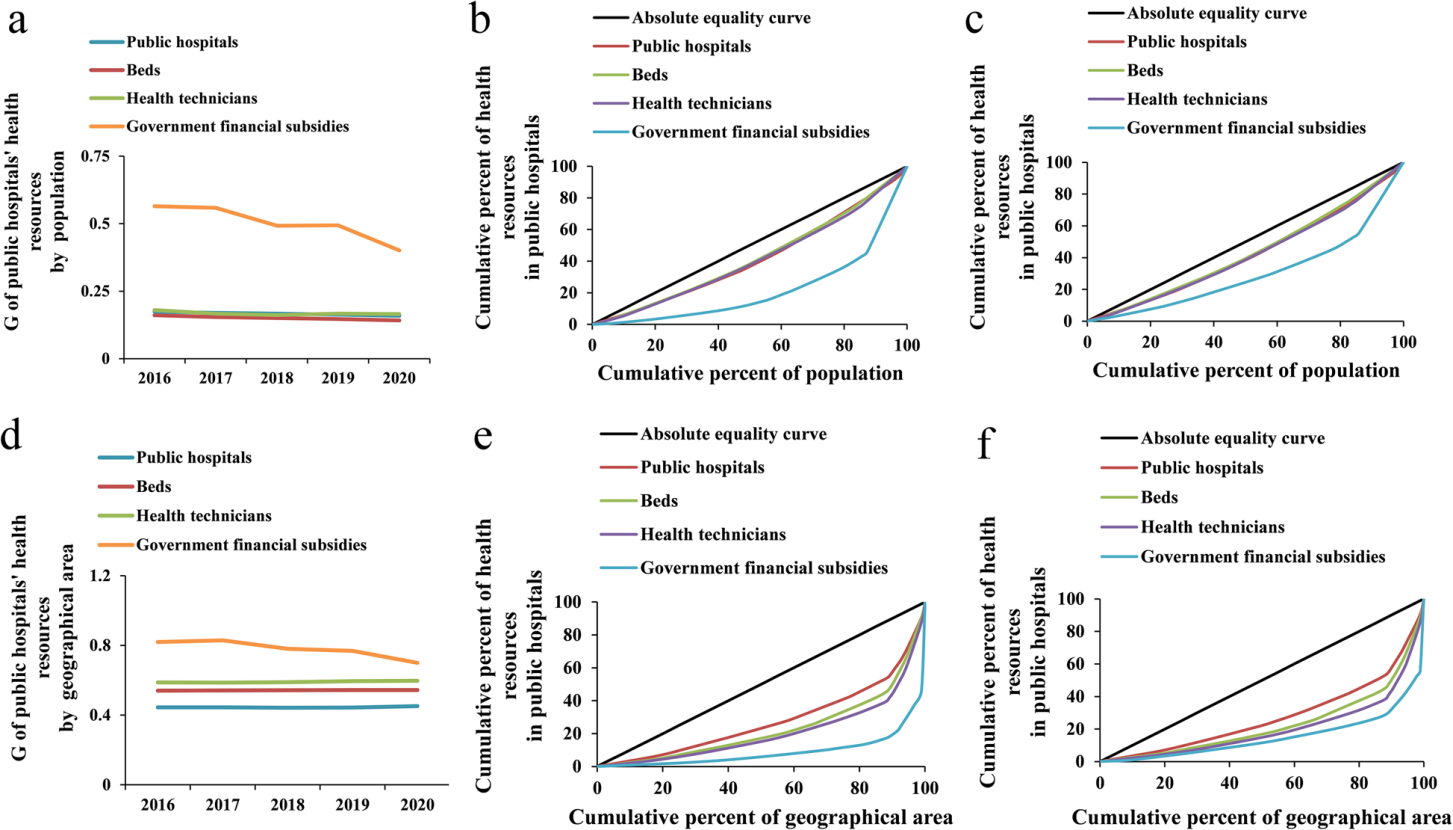
The equity of public hospitals’ health resource allocation in Guangdong Province from 2016 to 2020. **a** and **d** show the *G* of public hospitals’ health resources allocated by population and geographical area, respectively. **b** and **c** show the Lorenz curves of public hospitals’ health resources allocated by population in 2016 and 2020, respectively. **e** and **f** show the Lorenz curves of public hospitals’ health resources allocated by geographical area in 2016 and 2020, respectively

This study assesses the equity of public hospitals’ health resource distribution in terms of the demographic and geographical dimensions. For health resource allocation by population, the *Gs* of public hospitals, beds, and health technicians remained below 0.2, while the *Gs* of government financial subsidies were between 0.4 and 0.6 (Fig. 2a). For health resource allocation by geographical area, the *Gs* tended to grow, except for the government financial subsidies; moreover, the *Gs* of public hospitals, beds, and health technicians were all larger than 0.4, and that of government financial subsidies greater than 0.7, indicating an extreme level of unfairness (Fig. 2d). Only the Lorenz curves in 2016 and 2020 are presented here for representation given the limited space. Compared with e and f, the Lorenz curves in b and c were closer to the absolute equality curve, which means the public hospitals’ health resources were more equitable in terms of their allocation by population than by geographical area. What stands out in the figure is that the curves of government financial subsidies were the farthest from the absolute equality curve in terms of the demographic and geographical dimensions (Fig. 2b, c, e, f). The Lorenz curves in 2017, 2018, and 2019 present the same situation (detailed information can be seen in Fig. S1).

The *T* was calculated to measure the equity of public hospitals’ health resource allocation, and the results were consistent with those of the *G* and the Lorenz curves (Table 2). Further analysis of the sources showed that inequality came mainly from intraregional differences, with intraregional contribution rates of 52.97, 74.80, 61.95, and 83.19% for public hospitals, beds, health technicians, and government financial subsidies, respectively (Table 2). Subsequently, we continued to decompose the differences within regions. As shown in Table 3, the differences in the distribution of public hospitals, beds, health technicians, and government financial subsidies within the Pearl River Delta contributed 39.77, 55.20, 50.03, and 74.13% to the overall variation, respectively, which were greater than the contributions in the other three regions. To clarify the fairness of public hospitals’ health resource allocation in the four regions, *T* was computed for each region in Table 4. The *T* of beds, health technicians, and government financial subsidies was the largest in Pearl River Delta and the smallest in the western region, revealing that the allocation of beds, health technicians, and government financial subsidies is the most equitable in the western region and the least equitable in the Pearl River Delta. Additionally, government financial subsidies were the least equitable in all four regions.

**Table 2:** *T* of public hospitals’ health resource allocation in Guangdong Province from 2016 to 2020

Year	Theil index				Contribution rate of intraregion (%)				Contribution rate of interregion (%)			
	Public hospitals	Beds	Health technicians	Government financial subsidies	Public hospitals	Beds	Health technicians	Government financial subsidies	Public hospitals	Beds	Health technicians	Government financial subsidies
2016	0.0218	0.0179	0.0230	0.2591	55.88	73.22	56.67	53.93	44.12	26.78	43.33	46.07
2017	0.0204	0.0167	0.0200	0.2515	51.93	76.83	59.91	53.15	48.07	23.17	40.09	46.85
2018	0.0194	0.0160	0.0188	0.1816	49.97	77.19	61.25	62.63	50.03	22.81	38.75	37.37
2019	0.0184	0.0152	0.0198	0.1855	50.91	75.66	59.80	68.35	49.09	24.34	40.20	31.65
2020	0.0176	0.0141	0.0193	0.1177	52.97	74.80	61.95	83.19	47.03	25.20	38.05	16.81

**Table 3:** Contribution to the overall differences between regions from 2016 to 2020

Year	Public hospitals (%)				Beds (%)				Health technicians (%)				Government financial subsidies (%)			
	PRD	ER	WR	MR	PRD	ER	WR	MR	PRD	ER	WR	MR	PRD	ER	WR	MR
2016	40.35	1.49	3.72	10.32	56.91	5.90	0.02	10.39	46.26	3.36	1.27	5.78	48.06	1.35	2.71	1.80
2017	36.05	1.89	3.99	10.00	58.95	7.77	0.08	10.04	48.21	4.17	1.54	5.98	50.35	0.31	1.08	1.41
2018	36.37	1.80	3.36	8.44	58.95	7.77	0.08	10.04	49.30	4.36	1.35	6.24	58.67	0.47	0.08	3.41
2019	38.75	1.43	2.59	8.14	56.35	9.25	0.14	9.92	48.86	4.36	1.54	5.04	64.42	1.16	0.79	1.98
2020	39.77	1.40	2.80	9.00	55.20	9.07	0.17	10.37	50.03	4.70	1.96	5.26	74.13	2.47	2.38	4.20

*Note*: *PRD* Pearl River Delta, *ER* Eastern Region, *WR* Western Region, *MR* Mountainous Region

**Table 4:** *T* of public hospitals’ health resource allocation among different regions from 2016 to 2020

Year	Public hospitals				Beds				Health technicians				Government financial subsidies			
	PRD	ER	WR	MR	PRD	ER	WR	MR	PRD	ER	WR	MR	PRD	ER	WR	MR
2016	0.0147	0.0023	0.0062	0.0167	0.0171	0.0076	0.0000	0.0138	0.0179	0.0056	0.0022	0.0099	0.2088	0.0251	0.0542	0.0347
2017	0.0122	0.0028	0.0064	0.0155	0.0162	0.0095	0.0001	0.0127	0.0159	0.0061	0.0024	0.0091	0.2095	0.0057	0.0212	0.0269
2018	0.0115	0.0026	0.0051	0.0126	0.0152	0.0111	0.0001	0.0121	0.0152	0.0062	0.0020	0.0091	0.1743	0.0064	0.0011	0.0479
2019	0.0116	0.0020	0.0038	0.0118	0.0139	0.0107	0.0002	0.0118	0.0157	0.0066	0.0024	0.0078	0.1942	0.0165	0.0116	0.0287
2020	0.0113	0.0019	0.0039	0.0125	0.0126	0.0099	0.0002	0.0116	0.0156	0.0070	0.0030	0.0081	0.1407	0.0225	0.0224	0.0392

*Note*: *PRD* Pearl River Delta, *ER* Eastern Region, *WR* Western Region, *MR* Mountainous Region

**Fig. 3:**
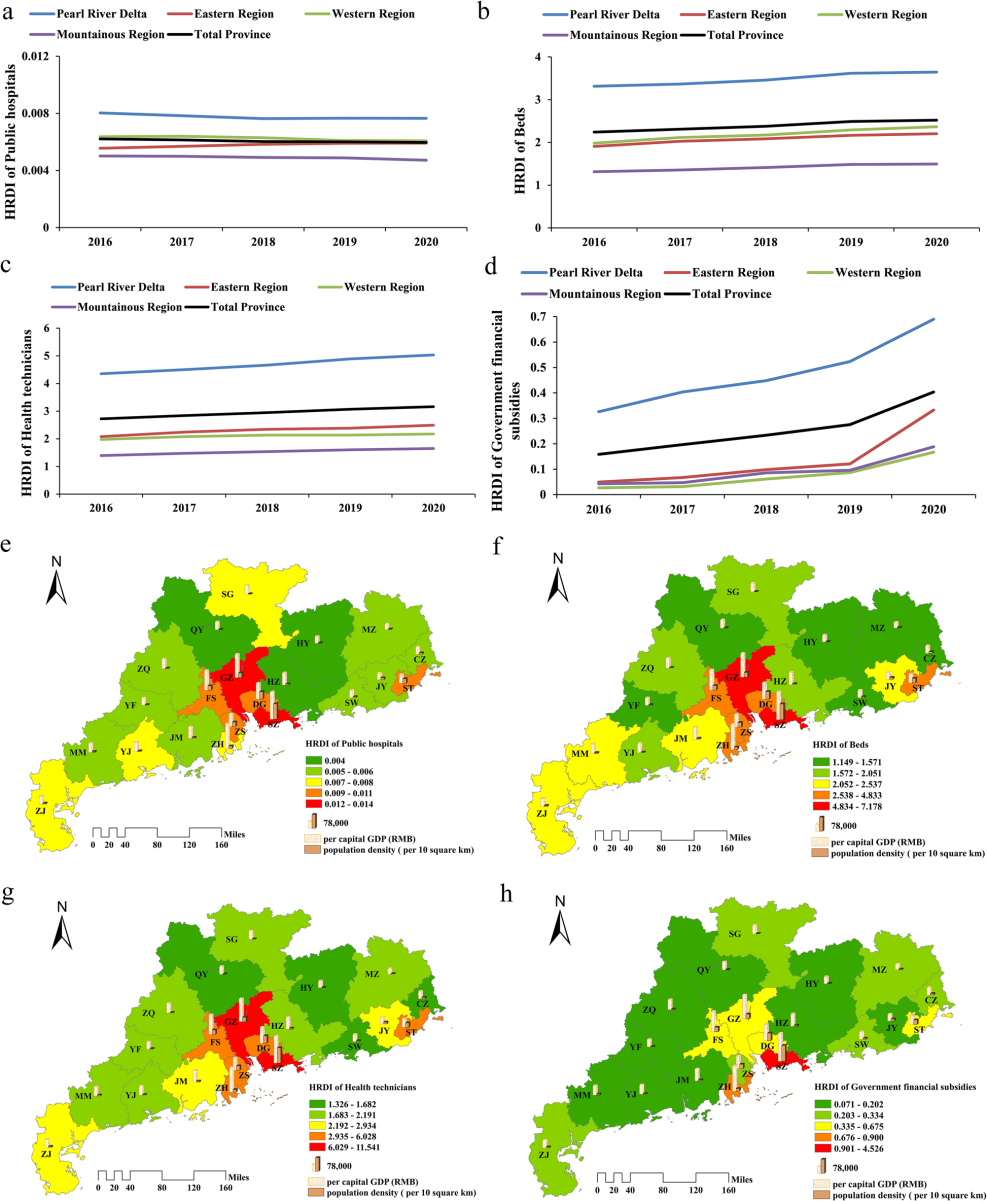
The HRDI of public hospitals’ health resources in Guangdong Province from 2016 to 2020. **a-d** denote the HRDI of public hospitals, beds and health technicians, and government financial subsidies in different regions. **e-h** denote the HRDI of public hospitals, beds and health technicians, and government financial subsidies in 21 cities

The HRDI was computed to analyze the equity of public hospitals’ health resource allocation by taking both demographic and geographical factors into consideration. It is apparent from the figure that the Pearl River Delta has the highest HRDI and is the only region that exceeds the provincial average (Fig. 3a-d). Additionally, the difference in the HRDI of beds, health technicians, and government financial subsidies between the Pearl River Delta and other regions of Guangdong Province is projected to increase. The spatial distribution of the HRDI in the data map was presented to vividly show the differences between the 21 cities in 2020 (Fig. 3e-h). As shown in the figure, there are at least four colors representing different levels of HRDI in the Pearl River Delta, which means the HRDI varies greatly between cities in the region. Specifically, GZ, SZ, DG, FS, ZH, and ZS have higher HRDI, while ZQ, HZ, and JM have lower HRDI. Notably, the cities with a high value of the HRDI always have relatively high per capita GDP and population density.

### The efficiency of public hospitals’ health resource allocation in Guangdong Province

Table 5 reported an increasing trend of beds and health technicians, while the number of public hospitals has decreased slightly in recent years. The outputs were on the rise from 2016 to 2019. However, they all decreased in 2020 due to the COVID-19 pandemic. The efficiency score and ranking of the cities was altered after deducting the influence of external environmental and random factors, and the likelihood ratio test rejected the null hypothesis at the 1% significant level (a comparison of the efficiency scores at stage 1 and stage 3 and the regression results of SFA are shown in Tables S2 and S3, respectively). This suggests that it is appropriate to use SFA model. According to Table 6, the adjusted efficiency score of public hospitals in Guangdong Province increased from 0.861 in 2016 to 0.915 in 2020, with an average value of 0.891. For specific regions, the mean efficiency scores are 0.999, 0.741, 0.889, and 0.819 for the Pearl River Delta, eastern region, western region and mountainous region, respectively. Five DEA-effective cities (23.81%) were found, including four in the Pearl River Delta and one in the western region in 2016. There were eight DEA-effective cities (38.1%) distributed in three regions, and most of them (six out of eight) were clustered in the Pearl River Delta Region in 2020.

**Table 5:** Descriptive statistics for inputs and outputs in Guangdong Province

Year	items	input			output			
		I_**1**_	I_**2**_	I_**3**_	O_**1**_	O_**2**_	O_**3**_	O_**4**_
2016	Mean	43.29	15,612	18,958	57	1795	116	9.876
	Maxi	160	69,877	96,500	249	9833	565	69.167
	Mini	14	3698	4306	14	277	10	1.578
2017	Mean	43.19	16,233	19,988	61	1816	127	10.848
	Maxi	152	71,339	99,642	270	9756	571	76.284
	Mini	14	3754	4465	14	280	10	1.699
2018	Mean	42.76	16,860	20,920	63	1827	137	12.062
	Maxi	145	73,905	103,450	287	9478	629	84.075
	Mini	14	3670	4447	14	300	13	1.818
2019	Mean	42.86	17,762	21,896	68	1952	147	13.823
	Maxi	145	77,664	109,343	309	10,191	660	96.436
	Mini	14	3769	4611	15	311	14	2.037
2020	Mean	42.76	18,072	22,666	58	1610	144	13.067
	Maxi	147	78,035	114,292	253	8041	602	88.544
	Mini	14	3924	4780	13	277	11	1.897

*Note*: I_1_: public hospitals, I_2_: beds, I_3_: health technicians, O_1_: number of discharges (ten thousand persons), O_2_: total number of visits (ten thousand persons), O_3_: number of health examinations (ten thousand persons), O_4_: annual medical revenue (billion RMB)

**Table 6:** Adjusted efficiency values of public hospitals’ health resource allocation in Guangdong Province

DMUs	2016	2017	2018	2019	2020
**PRD**	0.977	0.986	1.005	1.005	1.023
GZ	1.255	1.316	1.303	1.310	1.267
SZ	1.300	1.224	1.289	1.244	1.079
ZH	0.768	0.802	0.869	0.896	1.200
FS	1.121	1.087	1.089	1.104	1.103
HZ	0.851	0.857	0.879	0.881	0.859
DG	0.928	0.975	0.977	1.013	1.049
ZS	1.080	1.079	1.065	1.054	1.042
JM	0.858	0.862	0.883	0.858	0.894
ZQ	0.636	0.669	0.694	0.683	0.716
**ER**	0.714	0.722	0.757	0.756	0.756
ST	0.764	0.737	0.774	0.795	0.844
SW	0.623	0.639	0.660	0.635	0.614
CZ	0.654	0.673	0.674	0.672	0.663
JY	0.813	0.840	0.918	0.921	0.903
**WR**	0.852	0.853	0.905	0.921	0.915
YJ	0.713	0.716	0.781	0.787	0.725
ZJ	0.820	0.810	0.900	0.936	0.935
MM	1.023	1.031	1.035	1.039	1.085
**MR**	0.773	0.788	0.848	0.838	0.847
SG	0.785	0.799	0.854	0.861	0.870
HY	0.760	0.776	0.769	0.752	0.734
MZ	0.759	0.783	0.840	0.841	0.841
QY	0.838	0.877	1.010	1.005	1.005
YF	0.724	0.703	0.765	0.732	0.784
Mean	0.861	0.869	0.906	0.906	0.915
**Efficient DMUs**	5	5	6	7	8

*Note*: *PRD* Pearl River Delta, *ER* Eastern Region, *WR* Western Region, *MR* Mountainous Region

### Analysis of the coordination between the equity and efficiency of public hospitals’ health resource allocation

To further illustrate the balance between equity and efficiency in the allocation of health resources in public hospitals, two-dimensional diagrams were drawn to describe the distribution of the HRDI of the comprehensive indicator and efficiency, and government financial subsidies and efficiency. The HRDI of the comprehensive indicator is calculated to reflect the overall level of health resources occupation in each city according to geographical and demographic factors (weight of each indicator, comprehensive indicators, and the HRDI of the comprehensive indicators across cities are shown in Tables S4, S5 and S6). All cities and regions are divided into four quadrants in two-dimensional matrix diagrams, and the two red lines represent the average values of the 21 cities. Regions in the first quadrant have relatively adequate health resources and efficient use, regions in the second quadrant have relatively inadequate health resources but efficient use, regions in the third quadrant have relatively inadequate health resources and inefficient use, and regions in the fourth quadrant have relatively adequate health resources but inefficient use.

**Fig. 4:**
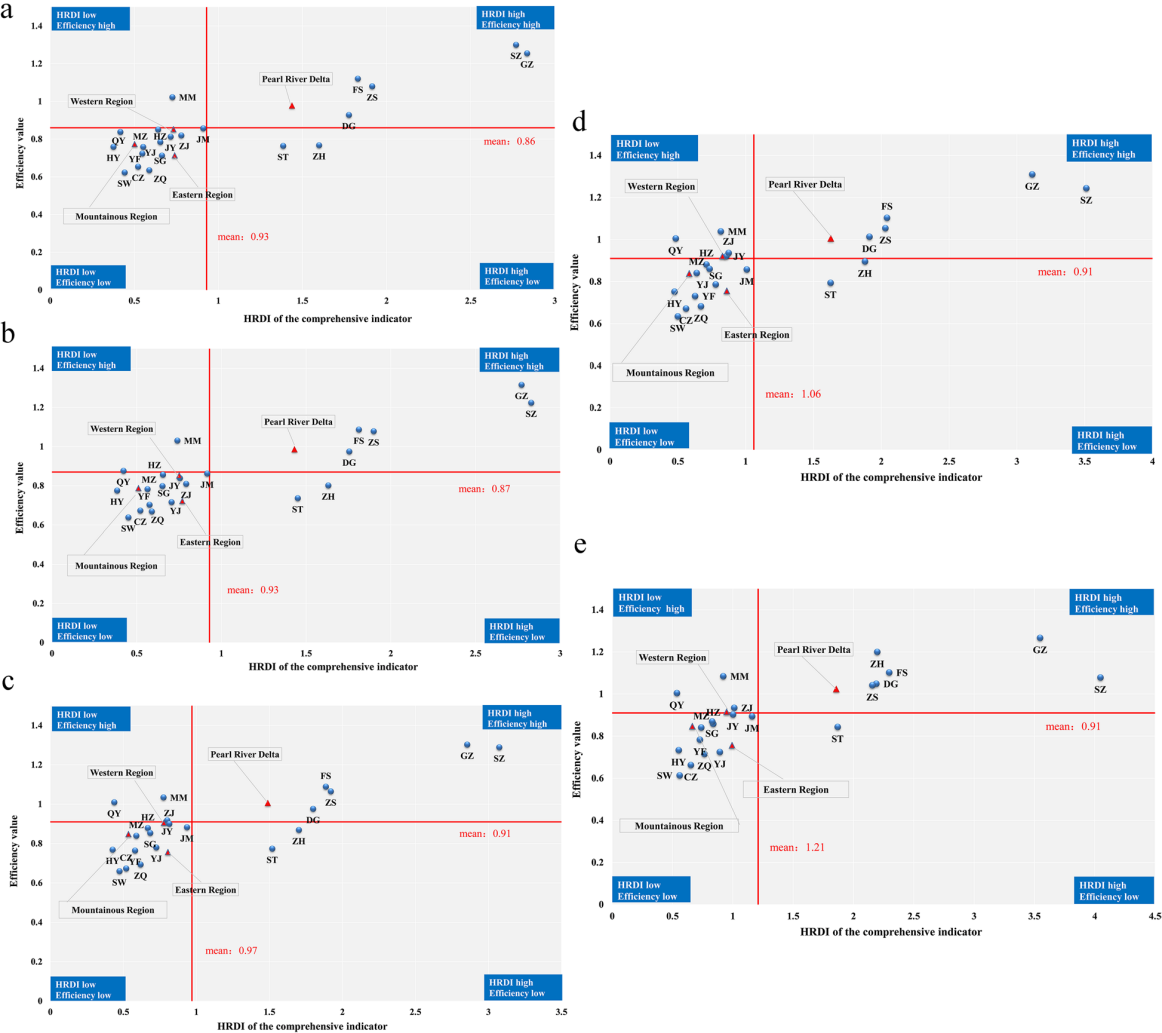
Distribution of the HRDI of the comprehensive indicator and efficiency from 2016 to 2020 (from **a** to **e**). The first to the fourth quadrants indicate high HRDI and high efficiency, low HRDI and high efficiency, low HRDI and low efficiency, and high HRDI and low efficiency, respectively

**Fig. 5:**
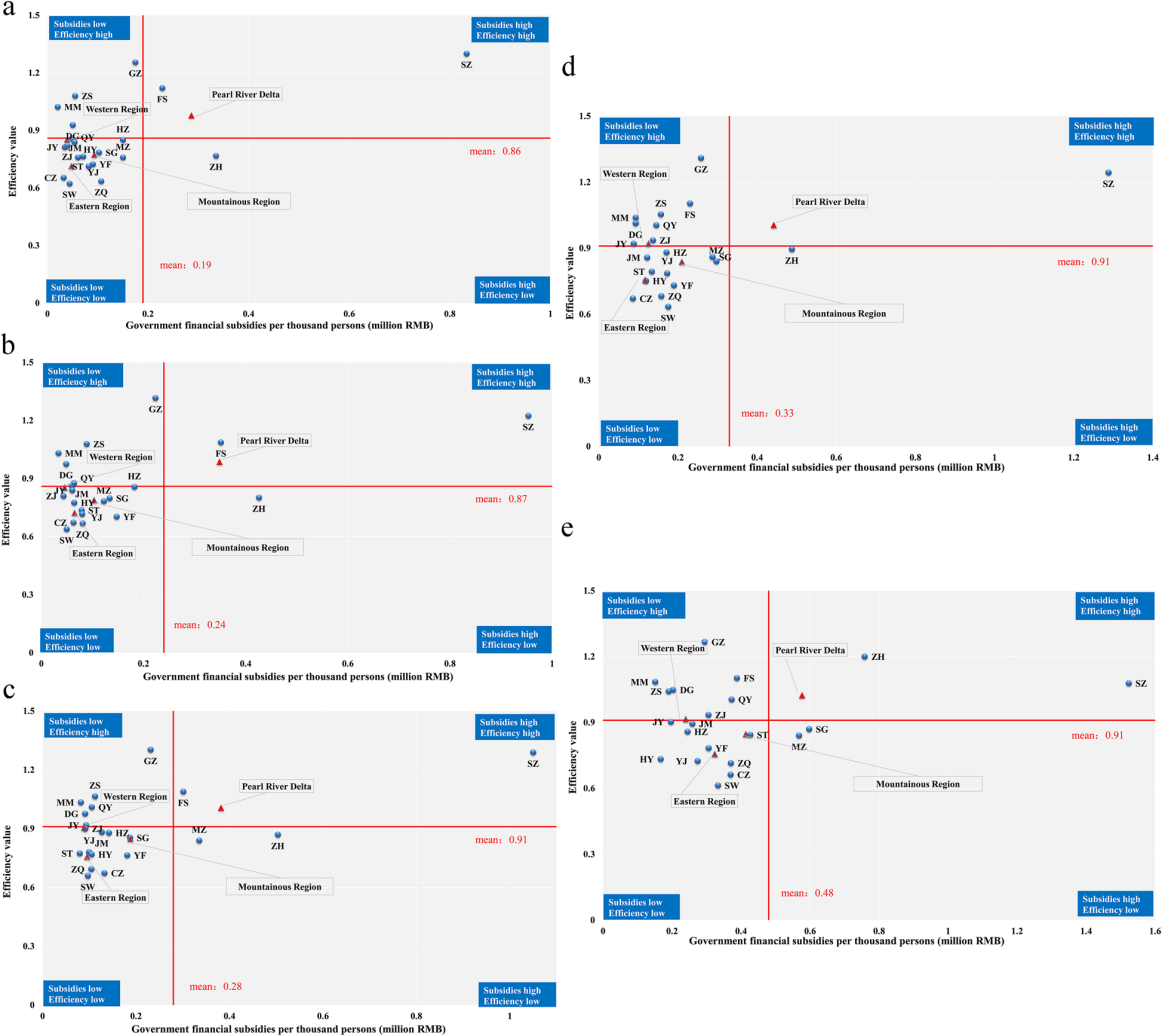
Distribution of government financial subsidies per thousand persons and efficiency from 2016 to 2020 (from **a** to **e**). The first to the fourth quadrants indicate high subsidies and high efficiency, low subsidies and high efficiency, low subsidies and low efficiency, and high subsidies and low efficiency, respectively

Figure 4 shows the distribution of the HRDI of the comprehensive indicator and efficiency from 2016 to 2020. As shown, the Pearl River Delta was in the first quadrant, and the other three regions were in the third quadrant during these five years. The number of cities in the first quadrant increased from 5 in the year of 2016 to 6 in the year of 2020, and all of them are located in the Pearl River Delta. On the other hand, the other three cities in the Pearl River Delta, HZ, JM, and ZQ, were in the third quadrant from 2016 to 2020. The number of cities in the third quadrant decreased, from 13 in 2016 to 10 in 2020. Cities in the second quadrant, such as MM and QY, had relatively high resource utilization efficiency but a relative lack of resources. ST was in the fourth quadrant from 2016 to 2020, indicating that it needs to improve the efficiency of health resource utilization based on the current number of health resources. The government’s financial subsidies to hospitals directly reflect the importance the government attaches to the development of health services. According to Fig. 5, approximately half of the 21 cities were in the third quadrant, which means that the government financial subsidies (per thousand persons) and efficiency values in these cities were below the provincial average.

## Discussion

During the five years from 2016 to 2020, the number of health resources and medical service capacity in public hospitals of Guangdong Province greatly improved. The study confirmed that beds, health technicians, and government financial subsidies showed an upward trend in total number, per thousand persons, and per square kilometer in Guangdong Province. In addition, the number of public hospitals decreased slightly, which is mainly due to the recent policies limiting the blind expansion of public hospitals in terms of quantity and scale to provide space for social capital to develop medical and health care and requiring public hospitals to pursue high-quality development of service and management [22].

This study analyzed the equity in the distribution of health resources, including public hospitals, beds, health technicians, and government financial subsidies, in Guangdong Province from 2016 to 2020. According to the Gini coefficients and Lorenz curves, the study reaches two conclusions. First, the distribution of public hospitals, beds, and health technicians by population is absolutely fair, while the distribution of government financial subsidies is unfair. This result is consistent with the finding of Li Q et al. [17]. The main reason why the government financial subsidies allocation is much less equitable than the allocation of the other three types of resources is that the amount of financial subsidies depends largely on the tax revenues of each city, which is deeply influenced by the level of local economic development. Additionally, a significant disparity in economic development between the Pearl River Delta, eastern region, western region, and mountainous region still exists [65]. Consequently, it is reasonable to suggest that the government improve the redistribution system and increase financial support for less-developed regions, covering the eastern region, western region, and mountainous region. Second, the fairness of health resources allocated by population is better than that allocated by geographic area, which is consistent with other studies on the equity of health resource distribution in Guangdong Province [66, 67]. There are two possible explanations for this result: first, some cities, such as SG, MZ, and HY, having a large geographical area, are backward in economic development and therefore face a lack of health resources; second, the health resource allocation standards set by the government are the number of health resources per thousand persons instead of the number of health resources per square kilometer [22]. However, it should not be ignored that the farther away residents are from the hospital, the more time and economic cost it takes to go to the hospital, and the less motivated residents will be to receive health services, which is not conducive to the construction of a healthy China. Thus, it is necessary for governments to take population and geographical factors into consideration when formulating health resource allocation plans [30, 51]. The results for *T* showed that the unfairness of health resource allocation in Guangdong Province mainly stemmed from intraregional differences. The Pearl River Delta has the largest variation in the internal distribution of health resources and contributes much more to the differences in the province than other regions. The same result was found in the study of Zhu C et al. [68]. Although the HRDI of the Pearl River Delta is far greater than that of the other three regions, the differences among cities in the Pearl River Delta are great, with the lowest values in JM, HZ, and ZQ. Taken together, these results suggest that the development of health services in the Pearl River Delta is extremely uneven, and improving the equity status of health resource allocation in the Pearl River Delta can effectively enhance the fairness of health resource allocation in the whole province. Therefore, it is highly recommended that the government attach great importance to the equity status of health resource distribution in the Pearl River Delta and ensure the basic medical and health needs of residents in underdeveloped and remote cities, including JM, HZ, and ZQ. It is equally important to promote the reasonable flow of medical experts, key personnel, and recent graduates of higher educational backgrounds from large cities to economically underdeveloped cities, with policy guidance and financial support, which could enable people living in rural, remote areas to have access to quality health resources and services.

After excluding the impact of external environmental and random factors, the efficiency scores and ranking of the cities changed. That is, environmental and random factors affect the real performance of hospitals. Therefore, it is reasonable and necessary to use the improved three-stage DEA method when measuring the efficiency of public hospitals for accurate results. The results for efficiency indicated that the mean efficiency score of public hospitals in Guangdong was 0.891, which was higher than that in Palestine (0.853) [32], Serbia (0.782) [45], Turkey (0.724) [69], Saudi Arabia (0.76) [50], Jordan (0.547) [70], and Iran (0.873) [71] and lower than that in Spain (1.016) [72], Wuhan, China (0.899) [73], and Taiwan, China (0.973) [74]. Only 38.1% of cities were found to be efficient in 2020. All these findings show that the results for the allocation of health resources in public hospitals in Guangdong Province were not satisfactory, and the health resources in most cities have not been fully utilized. On the other hand, cities found to be DEA-effective were mainly located in the Pearl River Delta, which indicates that it is of great significance to establish a mechanism for cooperation and exchange between the Pearl River Delta, eastern region, western region and mountainous region to encourage cities to learn from the advanced concepts and successful practices of cities at the forefront of efficiency in the Pearl River Delta, such as GZ, SZ, ZH, FS, DG, and ZS, to boost the efficiency in resource utilization. Although the government has been driving regional exchanges and cooperation [75], it seems that the effect is not obvious, the relevant system still needs to be improved, and related measures still need to be put in place.

For the distribution of the HRDI of the comprehensive indicator and efficiency, six out of nine cities in the Pearl River Delta were in the first quadrant, which means these cities have relatively sufficient health resources and high efficiency in resource utilization. However, the remaining three cities in the Pearl River Delta, HZ, JM, and ZQ, were in the third quadrant, indicating the simultaneous existence of a shortage of health resources and inefficient use of health resources. These results suggest that cities in the Pearl River Delta have polarized performance in the coordination of fairness and efficiency, which once again demonstrates that high priority should be given to the integrated development of health services in the Pearl River Delta to promote the sustainable development of healthcare in the region by strengthening regional health planning and focusing on less-developed cities, i.e., HZ, JM, and ZQ. The Pearl River Delta was in the first quadrant and the other three regions were in the third quadrant during these five years. In addition, cities in the third quadrant accounted for a large proportion of the total number of cities. Taken together, these indicate uneven development of health care between the four regions, and most cities in the eastern, western, and mountainous regions suffer the dual pressures of relatively insufficient health resources and inefficiency. Consequently, it is necessary to strengthen the cooperation and exchange between the Pearl River Delta, eastern, western and mountainous regions to promote the flow of health resources between regions, which is conducive to the improvement in hospital management and medical service in less-developed areas. In addition, cities in the third quadrant should learn from the health management and experience of cities including QY and MM in the second quadrant and emphasize improving efficiency in the case of limited health resources, enabling them to move from the third quadrant to the second quadrant and finally to the first quadrant. In this way, the dynamic coordination of equity and efficiency of health resource allocation can be realized.

## Limitations

Although we have attempted to use various methods and the improved three-stage DEA model to conduct a comprehensive assessment of the equity and efficiency of public hospitals’ health resource allocation, there are still some limitations to this study. First, the selection of indicators was based on previous studies, but like most studies, due to the availability of data, the output indicators did not include quality indicators, such as those related to patient health recovery, patient satisfaction, job satisfaction of medical staff, medication errors, etc. Second, although environmental factors were considered from five dimensions, i.e., economic development, population, education, insurance and policy, some alternative environmental variables that may also affect the efficiency scores should be incorporated in future studies. Third, when calculating the comprehensive index reflecting the overall level of health resources, the entropy weight method is first used to assign the weights of indicators. The entropy weight method is an objective method that can reduce the subjective influence of decision-makers and improve objectivity. However, future research can consider introducing subjective methods, such as Analytic Hierarchy Process and Delphi methods, to assign the weights of indicators, or combining the results of subjective and objective weighting methods.

## Conclusions

This study provides an empirical study of the equity and efficiency of public hospitals’ health resource allocation in Guangdong Province based on the most recent available data from official publications. The study has enlightenment for management and significance guidance for policymakers in formulating effective policies to realize the unification of equity and efficiency of health resource allocation. Major findings in the study and recommendations are as follows. With the development of the social economy, the total amount of health resources and the capacity of medical services greatly improved from 2016 to 2020 in Guangdong Province. The equity of government financial subsidies distribution was the worst, and there were distinct regional differences in the geographical distribution of public hospitals’ health resources in Guangdong Province. Furthermore, the development of health services in the Pearl River Delta was extremely uneven, which is reflected in the substantial differences in health resources allocation and resources utilization efficiency among cities in the region. The uneven development of health care between the Pearl River Delta, eastern region, western region, and mountainous region was also observed. Most cities in the eastern, western, and mountainous regions of Guangdong Province faced the problem of relatively insufficient health resources and low efficiency. To improve the equity and efficiency of health resource allocation, this study offers several suggestions. First, high priority should be given to the integrated development of health services in the Pearl River Delta, with a focus on key cities including HZ, JM, and ZQ. Second, demographic and geographical factors should be taken into consideration when formulating health resource allocation planning. Third, the government income redistribution system needs to be further improved, with more distribution to underdeveloped areas and more preferential policies to attract experienced medical workers and health technicians with a higher education background to work in the eastern region, western region and mountainous region. Finally, the cooperation and exchange between the Pearl River Delta, eastern region, western region and mountainous region should be strengthened to promote the flow of health resources between regions, increase the total amount of health resources and improve the medical service capacity in eastern region, western region and mountainous region.

## Supplementary Information

**Additional file 1: Table S1.** Public hospitals’ health resource allocation among different regions in Guangdong Province from 2016 to 2020.

**Additional file 2: Fig. S1.** Lorenz curves of public hospitals’ health resources from 2017 to 2019. a, b, and c show the Lorenz curves of public hospitals’ health resources allocated by population in 2017, 2018, and 2019, respectively. d, e, and f show the Lorenz curves of public hospitals’ health resources allocated by geographical area in 2017, 2018, and 2019, respectively.

**Additional file 3: Table S2.** Comparison of efficiency scores at stage 1 and stage 3.

**Additional file 4: Table S3.** Regression results of SFA in stage 2.

**Additional file 5: Table S4.** Weight of each indicator from 2016 to 2020.

**Additional file 6: Table S5.** Comprehensive indicators of 21 cities in Guangdong Province from 2016 to 2020.

**Additional file 7: Table S6.** The HRDI of the comprehensive indicators of 21 cities in Guangdong Province from 2016 to 2020.

## Data Availability

Please contact the authors for data request.

## Abbreviations

*G*: Gini coefficient
*T*: Theil index
HRDI: Health resource desity index
DEA: Data envelopment analysis
SBM: Slacks-based measure
GZ: Guangzhou
SZ: Shenzhen
DG: Dongguan
FS: Foshan
ZH: Zhuhai
ZS: Zhongshan
ZQ: Zhaoqing
HZ: Huizhou
JM: Jiangmen
ZJ: Zhanjiang
MM: Maoming
YJ: Yangjiang
YF: Yunfu
QY: Qingyuan
SG: Shaoguan
HY: Heyuan
MZ: Meizhou
SW: Shanwei
JY: Jieyang
ST: Shantou
CZ: Chaozhou
COVID-19: Coronavirus Disease 2019
GDP: Gross Domestic Product
DMUs: Decision-making units
CCR-DEA model: Charne, Cooper, Rhodes data envelopment analysis model
BCC-DEA model: Banker, Charnes, Cooper data envelopment analysis model
SFA: Stochastic frontier analysis

## References

[1] Li L (2021). On the reform of the public hospitals in the new era. Admin Reform.

[2] Yang YS, Liu Y (2019). Universal Medical Insurance and Social Governance: Exploration in the 70 Years after the Founding of the People's Republic of China. Admin Reform.

[3] Lin XH (2009). Equity and Efficiency of New Medical Reform. People's Polit Forum.

[4] Allen P, Cao Q, Wang H (2014). Public hospital autonomy in China in an international context. Int J Health Plann Manag.

[5] Mei J, Kirkpatrick I (2019). Public hospital reforms in China: towards a model of new public management?. Int J Public Sect Ma.

[6] Development Research Center of the State Council (2005). Evaluation and Recommendations on China's Medical and Health System Reform. China Health Policy.

[7] Department of Institutional Reform (2009). Deepen the reform of the medical and health system and realize the basic medical and health services for the population as soon as possible.

[8] Guangdong Provincial Health Ministry (2011). Guangdong Health Yearbook.

[9] Guangdong Provincial Health Ministry (2018). Guangdong Health Yearbook.

[10] Statistics Bureau of Guangdong Province (2022). Statistical Bulletin of National Economic and Social Development of Guangdong Province, 2021.

[11] National Bureau of Statistics (2022). Statistical Bulletin of the People's Republic of China on National Economic and Social Development, 2021.

[12] People's Government of Guangdong Province (2022). The 14th Five-Year Plan for the Development of Health undertakings in Guangdong Province.

[13] Ding J, Hu X, Zhang X, Shang L, Yu M, Chen H (2018). Equity and efficiency of medical service systems at the provincial level of China's mainland: a comparative study from 2009 to 2014. BMC Public Health.

[14] Yi M, Peng J, Zhang L, Zhang Y (2020). Is the allocation of medical and health resources effective? Characteristic facts from regional heterogeneity in China. Int J Equity Health.

[15] Pu L (2021). Fairness of the Distribution of Public Medical and Health Resources. Front Public Health.

[16] Dong E, Xu J, Sun X, Xu T, Zhang L, Wang T (2021). Differences in regional distribution and inequality in health-resource allocation on institutions, beds, and workforce: a longitudinal study in China. Arch Public Health.

[17] Li Q, Wei J, Jiang F, Zhou G, Jiang R, Chen M (2020). Equity and efficiency of health care resource allocation in Jiangsu Province, China. Int J Equity Health.

[18] Chen R, Zhao Y, Du J, Wu T, Huang Y, Guo A (2014). Health workforce equity in urban community health service of China. PLoS One.

[19] Xu K, Zhang K, Wang D, Zhou L (2014). Trend in distribution of primary health care professionals in Jiangsu province of eastern China. Int J Equity Health.

[20] Ma Y, Zhang L, Boswell M (2016). Inequities in the allocation of medical resources in China's Township Health Centers. China Agr Econ Rev.

[21] Wang S, Xu J, Jiang X, Li C, Li H, Song S (2018). Trends in health resource disparities in primary health care institutions in Liaoning Province in Northeast China. Int J Equity Health.

[22] Zhang Y, Wang Q, Jiang T, Wang J (2018). Equity and efficiency of primary health care resource allocation in mainland China. Int J Equity Health.

[23] Deng S, Zhang J, Teng X, Zhao Y (2022). Infectious disease reporting and management status in Anhui non-governmental medical institutions, 2019. Mod Prev Med.

[24] Peoples Network (2016). Number of private hospitals in China has exceeded that of public hospitals.

[25] National Health Commission of the People's Republic of China (2021). China Health Statistics Yearbook.

[26] National Health Commission of the People's Republic of China (2022). 2021 Statistical Bulletin on the Development of my country's Health and Wellness.

[27] Chen J, Tian X, Zhou B (2021). Comparative study on DEA efficiency between Public hospitals and Private hospitals in Shandong Province under the social medical system. Soft Sci Health.

[28] Kontodimopoulos N, Nanos P, Niakas D (2006). Balancing efficiency of health services and equity of access in remote areas in Greece. Health Policy.

[29] Bahrami MA, Rafiei S, Abedi M, Askari R (2018). Data envelopment analysis for estimating efficiency of intensive care units: a case study in Iran. Int J Health Care Qual Assur.

[30] Huang M, Luo D, Wang Z, Cao Y, Wang H, Bi F (2020). Equity and efficiency of maternal and child health resources allocation in Hunan Province, China. BMC Health Serv Res.

[31] Chitnis A, Mishra DK (2019). Performance Efficiency of Indian Private Hospitals Using Data Envelopment Analysis and Super-efficiency DEA. J Health Manag.

[32] Sultan WIM, Crispim J (2018). Measuring the efficiency of Palestinian public hospitals during 2010-2015: an application of a two-stage DEA method. BMC Health Serv Res.

[33] Jing R, Xu T, Lai X, Mahmoudi E, Fang H (2019). Technical Efficiency of Public and Private Hospitals in Beijing, China: A Comparative Study. Int J Environ Res Public Health.

[34] El Husseiny IA (2021). The efficiency of healthcare spending in lower-middle-income countries: an empirical investigation using a two-stage data envelopment analysis approach. Int J Healthc Techno.

[35] Lu W, Evans RD, Zhang T, Ni Z, Tao H (2020). Evaluation of resource utilization efficiency in obstetrics and gynecology units in China: A three-stage data envelopment analysis of the Shanxi province. Int J Health Plann Manag.

[36] Chen Z, Chen X, Gan X, Bai K, Baležentis T, Cui L (2020). Technical Efficiency of Regional Public Hospitals in China Based on the Three-Stage DEA. Int J Environ Res Public Health.

[37] Li Z, Si X, Ding Z, Li X, Zheng S, Wang Y (2021). Measurement and Evaluation of the Operating Efficiency of China's Basic Pension Insurance: Based on Three-Stage DEA Model. Risk Manag Healthc Policy.

[38] Fried HO, Lovell CAK, Schmidt SS, Yaisawarng S (2002). Accounting for environmental effects and statistical noise in data envelopment analysis. J Prod Anal.

[39] Tone K (2002). A slacks-based measure of super-efficiency in data envelopment analysis. Eur J Oper Res.

[40] Kohl S, Schoenfelder J, Fügener A, Brunner JO (2019). The use of Data Envelopment Analysis (DEA) in healthcare with a focus on hospitals. Health Care Manag Sci.

[41] İlgün G, Sönmez S, Konca M, Yetim B (2022). Measuring the efficiency of Turkish maternal and child health hospitals: A two-stage data envelopment analysis. Eval Program Plann.

[42] Li Z, Yang L, Tang S, Bian Y (2020). Equity and Efficiency of Health Resource Allocation of Chinese Medicine in Mainland China: 2013-2017. Front Public Health.

[43] Ng YC (2011). The productive efficiency of Chinese hospitals. China Econ Rev.

[44] Sultan WIM, Crispim J (2018). Are public hospitals reforming efficiently in West Bank?. Confl Heal.

[45] Medarević A, Vuković D (2021). Efficiency and Productivity of Public Hospitals in Serbia Using DEA-Malmquist Model and Tobit Regression Model, 2015-2019. Int J Environ Res Public Health.

[46] Xu X, Li W, Tang L, Tang L, Xu X (2021). Study on the Efficiency of Health Resource Allocation in China-Based on Three-stage DEA Model. Health Econ Res.

[47] Zhao K, Ma S (2021). Analysis on the allocation efficiency of health resources at the grassroots level in various regions of China based on DEA. Chin Hosp.

[48] Cai X, Zhang Q, Wang X (2022). Evaluation on Allocation Efficiency of Medical and Health Resources in Shanghai Before and After New Medical Reform. Med Soc.

[49] Gan M, Zhang X (2021). Evaluation of the Efficiency of Health Resource Allocation of TCM Medical Institution in China Based on DEA and SFA. Chin Health Serv Manage.

[50] Alatawi AD, Niessen LW, Khan JAM (2020). Efficiency evaluation of public hospitals in Saudi Arabia: an application of data envelopment analysis. BMJ Open.

[51] Sun J, Luo H (2017). Evaluation on equality and efficiency of health resources allocation and health services utilization in China. Int J Equity Health.

[52] Hu H, Qi Q, Yang C (2012). Analysis of hospital technical efficiency in China: Effect of health insurance reform. China Econ Rev.

[53] National Bureau of Statistics (2021). Guangdong Statistical Yearbook.

[54] Li W, Han CX (2020). Study on the Equity of GP in Western Areas——Based on Gini Coefficient and Concentration. Health Econ Res.

[55] Huang Z, Feng Z, Zhang C (2016). Equity of health resources allocation in Hainan Province: an evaluation with Theil index. Chin J Public Health.

[56] Zhang N (2014). Analyzing the equity of health resources allocation in China based on Theil Index. Chin Health Serv Manage.

[57] Shannon CE (1997). The mathematical theory of communication (Reprinted). MD Comput.

[58] Su Y, Yang J, Si M, Li S, Tang R (2017). Study on Ningxia Health Resource Allocation Based on Entropy Weight Index of Resource Distribution. Chin Health Econ.

[59] Lee HC, Chang CT (2018). Comparative analysis of MCDM methods for ranking renewable energy sources in Taiwan. Renew Sust Energ Rev.

[60] Liang L, Wang Z, Li J (2019). The effect of urbanization on environmental pollution in rapidly developing urban agglomerations. J Clean Prod.

[61] Li X, Wang Z, Zhang L, Zou C, Dorrell DD (2019). State-of-health estimation for Li-ion batteries by combing the incremental capacity analysis method with grey relational analysis. J Power Sources.

[62] Li Z, Qiao X, Yang Y, Ding H, Zhang Y, Lu C (2022). Quality Evaluation of Angelicae Dahuricae Radix Decoction Pieces by Entropy Weight and Gray Relative Ananlysis Method. Chin J Mod Appl Pharmacy.

[63] Wang B, Chen M, Zheng S (2022). Comprehensive Evaluation of Inter-regional Scientific and Technological Innovation Capability in Anhui Province Based on AHP-EWM Method. Sci Technol Manage Res.

[64] Luo D (2012). A Note on Estimating Managerial Inefficiency of Three-stage DEA Model. Stat Res.

[65] China Business Network (2018). The growth rate of the Pearl River Delta leading Guangdong, the gap between the Pearl River Delta and other regions widening.

[66] Ye B, Hu W, Feng L, Xie Y, Huang X (2020). The analysis of equity in the allocation of health resources in Guangdong province. Modern Hospital.

[67] Zhang M, Lan S, Wei J, Luo Q, Li L, Liang H (2018). Analysis on the fairness of physician resources allocation in Guangdong province before and after the new medical reform. Health Econ Res.

[68] Zhu C, Li Q (2020). Analyzing the Equity of Health Resource Allocation in Guangdong Province. Chin Prim Health Care.

[69] Cinaroglu S (2020). Integrated k-means clustering with data envelopment analysis of public hospital efficiency. Health Care Manag Sci.

[70] Alwaked AA, Al-qalawi UR, Azaizeh SY. Efficiency of Jordanian public hospitals (2006-2015). J Public Aff. 2022;22:e2383.

[71] Kakemam E, Dargahi H (2019). The Health Sector Evolution Plan and the Technical Efficiency of Public Hospitals in Iran. Iran J Public Health.

[72] Fuentes R, Ferrándiz-Gomis R, Fuster-Garcia B (2019). Efficiency of acute public hospitals in the region of Murcia, Spain. J Comp Eff Res.

[73] Yin G, Chen C, Zhuo L, He Q, Tao H (2021). Efficiency Comparison of Public Hospitals under Different Administrative Affiliations in China: A Pilot City Case. Healthcare (Basel).

[74] Lin CS, Chiu CM, Huang YC, Lang HC, Chen MS (2021). Evaluating the Operational Efficiency and Quality of Tertiary Hospitals in Taiwan: The Application of the EBITDA Indicator to the DEA Method and TOBIT Regression. Healthcare (Basel).

[75] People's Political Consultative Conference Network: The Guangdong Provincial Committee of the Chinese People's Political Consultative Conference research the Pearl River Delta to help the Eastern, Western, and Mountainous Regions with medical and health poverty alleviation. (2016). http://www.rmzxb.com.cn/c/2016-06-16/870194.shtml. Accessed 12 Jun 2022.

