# Supplementary material for: Equity and efficiency of public hospitals’ health resource allocation in Guangdong Province, China
Source: Int J Equity Health. 2022 Sep 22;21:138. doi: 10.1186/s12939-022-01741-1 (PMC9493174; doi:10.1186/s12939-022-01741-1)
Supplement: Supplementary file 1 — Additional file 1: Table S1. Public hospitals’ health resource allocation among different regions in Guangdong Province from 2016 to 2020. [file 12939_2022_1741_MOESM1_ESM.docx]

**Additional file 1: Table S1.** Public hospitals’ health resource allocation among different regions in Guangdong Province from 2016 to 2020

| **Year** | **Region** | **Public hospitals** | | |  | **Beds** | | |  | **Health Technicians** | | |  | **Government financial subsidies(million)** | | |
| --- | --- | --- | --- | --- | --- | --- | --- | --- | --- | --- | --- | --- | --- | --- | --- | --- |
|  |  | **/1000 persons** | **/km^2^** | **Proportion (%)** |  | **/1000 persons** | **/km^2^** | **Proportion (%)** |  | **/1000 persons** | **/km^2^** | **Proportion (%)** |  | **/1000 persons** | **/km^2^** | **Proportion (%)** |
| 2016 | PRD | 0.0071 | 0.0091 | 55.12 |  | 2.9100 | 3.7734 | 63.03 |  | 3.8239 | 4.9584 | 68.21 |  | 0.2862 | 0.3712 | 87.55 |
|  | ER | 0.0054 | 0.0058 | 9.79 |  | 1.8427 | 1.9735 | 9.32 |  | 2.0072 | 2.1497 | 8.36 |  | 0.0477 | 0.0511 | 3.41 |
|  | WR | 0.0092 | 0.0044 | 15.73 |  | 2.8819 | 1.3650 | 13.59 |  | 2.8746 | 1.3615 | 11.16 |  | 0.0391 | 0.0185 | 2.60 |
|  | MR | 0.0110 | 0.0023 | 19.36 |  | 2.8754 | 0.6006 | 14.06 |  | 3.0477 | 0.6366 | 12.27 |  | 0.0933 | 0.0195 | 6.44 |
| 2017 | PRD | 0.0068 | 0.0091 | 54.80 |  | 2.9071 | 3.8949 | 62.58 |  | 3.8890 | 5.2105 | 67.99 |  | 0.3488 | 0.4674 | 88.17 |
|  | ER | 0.0055 | 0.0059 | 10.03 |  | 1.9616 | 2.0911 | 9.49 |  | 2.1730 | 2.3164 | 8.54 |  | 0.0652 | 0.0695 | 3.70 |
|  | WR | 0.0093 | 0.0044 | 15.88 |  | 3.0691 | 1.4596 | 13.98 |  | 3.0119 | 1.4324 | 11.14 |  | 0.0455 | 0.0216 | 2.43 |
|  | MR | 0.0109 | 0.0023 | 19.29 |  | 2.9707 | 0.6195 | 13.95 |  | 3.2340 | 0.6744 | 12.33 |  | 0.1034 | 0.0216 | 5.70 |
| 2018 | PRD | 0.0065 | 0.0090 | 54.68 |  | 2.9455 | 4.0579 | 62.77 |  | 3.9738 | 5.4746 | 68.25 |  | 0.3820 | 0.5263 | 82.87 |
|  | ER | 0.0057 | 0.0060 | 10.36 |  | 2.0226 | 2.1481 | 9.39 |  | 2.2765 | 2.4178 | 8.52 |  | 0.0953 | 0.1012 | 4.50 |
|  | WR | 0.0091 | 0.0043 | 15.81 |  | 3.1425 | 1.5030 | 13.86 |  | 3.0889 | 1.4774 | 10.98 |  | 0.0890 | 0.0425 | 3.99 |
|  | MR | 0.0108 | 0.0022 | 19.15 |  | 3.0984 | 0.6449 | 13.98 |  | 3.3692 | 0.7012 | 12.25 |  | 0.1880 | 0.0391 | 8.63 |
| 2019 | PRD | 0.0065 | 0.0091 | 55.22 |  | 3.0538 | 4.2844 | 62.91 |  | 4.1294 | 5.7934 | 69.01 |  | 0.4420 | 0.6201 | 82.43 |
|  | ER | 0.0057 | 0.0061 | 10.44 |  | 2.1035 | 2.2289 | 9.25 |  | 2.3190 | 2.4573 | 8.27 |  | 0.1173 | 0.1243 | 4.67 |
|  | WR | 0.0088 | 0.0042 | 15.33 |  | 3.3054 | 1.5891 | 13.91 |  | 3.0827 | 1.4821 | 10.52 |  | 0.1255 | 0.0603 | 4.78 |
|  | MR | 0.0107 | 0.0022 | 19.00 |  | 3.2575 | 0.6772 | 13.94 |  | 3.5163 | 0.7310 | 12.20 |  | 0.2098 | 0.0436 | 8.12 |
| 2020 | PRD | 0.0064 | 0.0091 | 55.79 |  | 3.0491 | 4.3554 | 62.85 |  | 4.2094 | 6.0128 | 69.19 |  | 0.5771 | 0.8243 | 74.26 |
|  | ER | 0.0058 | 0.0061 | 10.47 |  | 2.1452 | 2.2620 | 9.22 |  | 2.4270 | 2.5591 | 8.32 |  | 0.3237 | 0.3413 | 8.69 |
|  | WR | 0.0088 | 0.0042 | 15.37 |  | 3.4078 | 1.6458 | 14.16 |  | 3.1283 | 1.5108 | 10.36 |  | 0.2397 | 0.1158 | 6.22 |
|  | MR | 0.0104 | 0.0021 | 18.37 |  | 3.2815 | 0.6806 | 13.76 |  | 3.6268 | 0.7523 | 12.13 |  | 0.4136 | 0.0858 | 10.83 |

Note: PRD: Pearl River Delta; Eastern Region: ER; Western Region: WR; Mountainous Region: MR
