# Supplementary material for: Equity and efficiency of public hospitals’ health resource allocation in Guangdong Province, China
Source: Int J Equity Health. 2022 Sep 22;21:138. doi: 10.1186/s12939-022-01741-1 (PMC9493174; doi:10.1186/s12939-022-01741-1)
Supplement: Supplementary file 2 — Additional file 2: Fig. S1. Lorenz curves of public hospitals’ health resources from 2017 to 2019. a, b, and c show the Lorenz curves of public hospitals’ health resources allocated by population in 2017, 2018, and 2019, respectively. d, e, and f show the Lorenz curves of public hospitals’ health resources allocated by geographical area in 2017, 2018, and 2019, respectively. [file 12939_2022_1741_MOESM2_ESM.docx]

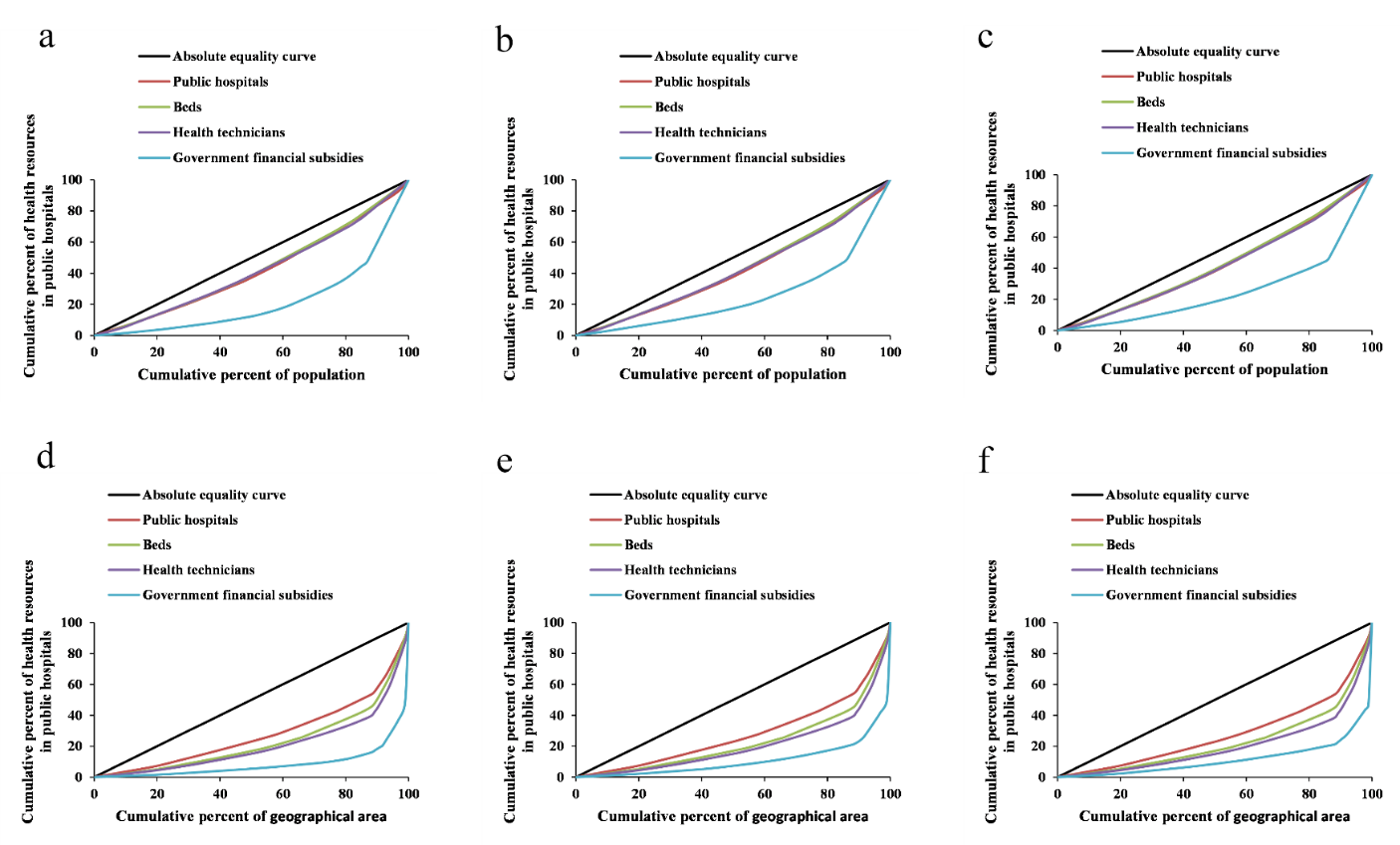


**Additional file 2: Fig. S1.** Lorenz curves of public hospitals’ health resources from 2017 to 2019. a, b, and c show the Lorenz curves of public hospitals’ health resources allocated by population in 2017, 2018, and 2019, respectively. d, e, and f show the Lorenz curves of public hospitals’ health resources allocated by geographical area in 2017, 2018, and 2019, respectively
