# Supplementary material for: Equity and efficiency of public hospitals’ health resource allocation in Guangdong Province, China
Source: Int J Equity Health. 2022 Sep 22;21:138. doi: 10.1186/s12939-022-01741-1 (PMC9493174; doi:10.1186/s12939-022-01741-1)
Supplement: Supplementary file 3 — Additional file 3: Table S2. Comparison of efficiency scores at stage 1 and stage 3. [file 12939_2022_1741_MOESM3_ESM.docx]

**Additional file 3: Table S2.** Comparison of efficiency scores at stage 1 and stage 3

| **Year** | **DMUs** | **Stage 1** | |  | **Stage 3** | |
| --- | --- | --- | --- | --- | --- | --- |
|  |  | **efficiency score** | **rank** |  | **efficiency score** | **rank** |
| 2016 | GZ | 1.222 | 2 |  | 1.255 | 2 |
|  | SG | 0.746 | 14 |  | 0.785 | 12 |
|  | SZ | 1.242 | 1 |  | 1.300 | 1 |
|  | ZH | 1.016 | 6 |  | 0.768 | 13 |
|  | ST | 0.740 | 16 |  | 0.764 | 14 |
|  | FS | 1.069 | 4 |  | 1.121 | 3 |
|  | JM | 0.806 | 9 |  | 0.858 | 7 |
|  | ZJ | 0.742 | 15 |  | 0.820 | 10 |
|  | MM | 1.017 | 5 |  | 1.023 | 5 |
|  | ZQ | 0.590 | 21 |  | 0.636 | 20 |
|  | HZ | 0.770 | 12 |  | 0.851 | 8 |
|  | MZ | 0.716 | 17 |  | 0.759 | 16 |
|  | SW | 0.712 | 18 |  | 0.623 | 21 |
|  | HY | 0.788 | 10 |  | 0.760 | 15 |
|  | YJ | 0.703 | 19 |  | 0.713 | 18 |
|  | QY | 1.003 | 7 |  | 0.838 | 9 |
|  | DG | 0.887 | 8 |  | 0.928 | 6 |
|  | ZS | 1.119 | 3 |  | 1.080 | 4 |
|  | CZ | 0.750 | 13 |  | 0.654 | 19 |
|  | JY | 0.788 | 11 |  | 0.813 | 11 |
|  | YF | 0.701 | 20 |  | 0.724 | 17 |
| 2017 | GZ | 1.282 | 1 |  | 1.316 | 1 |
|  | SG | 0.751 | 13 |  | 0.799 | 13 |
|  | SZ | 1.178 | 2 |  | 1.224 | 2 |
|  | ZH | 1.014 | 6 |  | 0.802 | 12 |
|  | ST | 0.703 | 17 |  | 0.737 | 16 |
|  | FS | 1.035 | 4 |  | 1.087 | 3 |
|  | JM | 0.804 | 10 |  | 0.862 | 8 |
|  | ZJ | 0.731 | 15 |  | 0.810 | 11 |
|  | MM | 1.026 | 5 |  | 1.031 | 5 |
|  | ZQ | 0.613 | 21 |  | 0.669 | 20 |
|  | HZ | 0.768 | 12 |  | 0.857 | 9 |
|  | MZ | 0.729 | 16 |  | 0.783 | 14 |
|  | SW | 0.702 | 18 |  | 0.639 | 21 |
|  | HY | 0.775 | 11 |  | 0.776 | 15 |
|  | YJ | 0.681 | 20 |  | 0.716 | 17 |
|  | QY | 0.896 | 8 |  | 0.877 | 7 |
|  | DG | 0.934 | 7 |  | 0.975 | 6 |
|  | ZS | 1.132 | 3 |  | 1.079 | 4 |
|  | CZ | 0.735 | 14 |  | 0.673 | 19 |
|  | JY | 0.816 | 9 |  | 0.840 | 10 |
|  | YF | 0.686 | 19 |  | 0.703 | 18 |
| 2018 | GZ | 1.270 | 1 |  | 1.303 | 1 |
|  | SG | 0.811 | 13 |  | 0.854 | 13 |
|  | SZ | 1.199 | 2 |  | 1.289 | 2 |
|  | ZH | 1.043 | 4 |  | 0.869 | 12 |
|  | ST | 0.770 | 15 |  | 0.774 | 16 |
|  | FS | 1.037 | 5 |  | 1.089 | 3 |
|  | JM | 0.842 | 10 |  | 0.883 | 10 |
|  | ZJ | 0.819 | 12 |  | 0.900 | 9 |
|  | MM | 1.029 | 6 |  | 1.035 | 5 |
|  | ZQ | 0.651 | 21 |  | 0.694 | 19 |
|  | HZ | 0.828 | 11 |  | 0.879 | 11 |
|  | MZ | 0.795 | 14 |  | 0.840 | 14 |
|  | SW | 0.728 | 20 |  | 0.660 | 21 |
|  | HY | 0.769 | 16 |  | 0.769 | 17 |
|  | YJ | 0.767 | 17 |  | 0.781 | 15 |
|  | QY | 1.028 | 7 |  | 1.010 | 6 |
|  | DG | 0.917 | 8 |  | 0.977 | 7 |
|  | ZS | 1.108 | 3 |  | 1.065 | 4 |
|  | CZ | 0.762 | 19 |  | 0.674 | 20 |
|  | JY | 0.904 | 9 |  | 0.918 | 8 |
|  | YF | 0.764 | 18 |  | 0.765 | 18 |
| 2019 | GZ | 1.278 | 1 |  | 1.310 | 1 |
|  | SG | 0.861 | 12 |  | 0.861 | 12 |
|  | SZ | 1.093 | 2 |  | 1.244 | 2 |
|  | ZH | 1.070 | 4 |  | 0.896 | 10 |
|  | ST | 0.832 | 15 |  | 0.795 | 15 |
|  | FS | 1.051 | 5 |  | 1.104 | 3 |
|  | JM | 0.857 | 13 |  | 0.858 | 13 |
|  | ZJ | 0.895 | 10 |  | 0.936 | 8 |
|  | MM | 1.043 | 6 |  | 1.039 | 5 |
|  | ZQ | 0.680 | 21 |  | 0.683 | 19 |
|  | HZ | 0.867 | 11 |  | 0.881 | 11 |
|  | MZ | 0.852 | 14 |  | 0.841 | 14 |
|  | SW | 0.741 | 20 |  | 0.635 | 21 |
|  | HY | 0.809 | 18 |  | 0.752 | 17 |
|  | YJ | 0.832 | 16 |  | 0.787 | 16 |
|  | QY | 1.028 | 7 |  | 1.005 | 7 |
|  | DG | 1.004 | 8 |  | 1.013 | 6 |
|  | ZS | 1.090 | 3 |  | 1.054 | 4 |
|  | CZ | 0.817 | 17 |  | 0.672 | 20 |
|  | JY | 0.966 | 9 |  | 0.921 | 9 |
|  | YF | 0.788 | 19 |  | 0.732 | 18 |
| 2020 | GZ | 1.239 | 2 |  | 1.267 | 1 |
|  | SG | 0.850 | 13 |  | 0.870 | 12 |
|  | SZ | 1.011 | 7 |  | 1.079 | 5 |
|  | ZH | 1.422 | 1 |  | 1.200 | 2 |
|  | ST | 0.860 | 12 |  | 0.844 | 14 |
|  | FS | 1.043 | 5 |  | 1.103 | 3 |
|  | JM | 0.891 | 10 |  | 0.894 | 11 |
|  | ZJ | 0.888 | 11 |  | 0.935 | 9 |
|  | MM | 1.090 | 3 |  | 1.085 | 4 |
|  | ZQ | 0.696 | 21 |  | 0.716 | 19 |
|  | HZ | 0.845 | 15 |  | 0.859 | 13 |
|  | MZ | 0.841 | 16 |  | 0.841 | 15 |
|  | SW | 0.698 | 20 |  | 0.614 | 21 |
|  | HY | 0.764 | 18 |  | 0.734 | 17 |
|  | YJ | 0.739 | 19 |  | 0.725 | 18 |
|  | QY | 1.020 | 6 |  | 1.005 | 8 |
|  | DG | 0.968 | 8 |  | 1.049 | 6 |
|  | ZS | 1.083 | 4 |  | 1.042 | 7 |
|  | CZ | 0.785 | 17 |  | 0.663 | 20 |
|  | JY | 0.929 | 9 |  | 0.903 | 10 |
|  | YF | 0.846 | 14 |  | 0.784 | 16 |
