# Supplementary material for: Equity and efficiency of public hospitals’ health resource allocation in Guangdong Province, China
Source: Int J Equity Health. 2022 Sep 22;21:138. doi: 10.1186/s12939-022-01741-1 (PMC9493174; doi:10.1186/s12939-022-01741-1)
Supplement: Supplementary file 4 — Additional file 4: Table S3. Regression results of SFA in stage 2. [file 12939_2022_1741_MOESM4_ESM.docx]

**Additional file 4: Table S3.** Regression results of SFA in stage 2

| **Environmental Variables** | **Public hospitals** | |  | **Beds** | |  | **health technicians** | |
| --- | --- | --- | --- | --- | --- | --- | --- | --- |
|  | **coefficient** | **t-test** |  | **coefficient** | **t-test** |  | **coefficient** | **t-test** |
| Constant | -1.348 | -0.169 |  | -2519.912^**^ | -4.088 |  | -1313.361 | -1.385 |
| Population density | -0.001 | -0.748 |  | -0.288^**^ | -2.370 |  | -0.229^*^ | -2.048 |
| Per capita GDP | 0.000 | -0.168 |  | -0.005 | -0.890 |  | -0.004 | -0.663 |
| Urbanization rate | -0.032 | -0.460 |  | 8.392 | 0.930 |  | -0.594 | -0.051 |
| Proportion of high school graduates | -199.941 | -0.478 |  | 3042.064^**^ | 368.937 |  | -37251.776^**^ | -522.925 |
| Proportion of people with basic medical insurance | 1.843 | 0.709 |  | 1254.584^**^ | 2.420 |  | 1057.079^**^ | 2.782 |
| Proportion of financial expenditure on health | 0.313 | 1.496 |  | 100.234^**^ | 3.290 |  | 70.672^*^ | 1.931 |
| Sigma-squared | 25.714^**^ | 2.853 |  | 2568231.200^**^ | 2020748.300 |  | 2422639.200^**^ | 1299197.500 |
| γ | 0.810^**^ | 11.139 |  | 0.939^**^ | 106.001 |  | 0.952^**^ | 153.570 |
| Log likelihood | -260.307 | |  | -812.844 | |  | -796.032 | |
| One-sided LR Test | 87.282^**^ | |  | 129.436^**^ | |  | 153.654^**^ | |

Note: *, ** denote significance at the 5% and 1% level, respectively.
