# Supplementary material for: Equity and efficiency of public hospitals’ health resource allocation in Guangdong Province, China
Source: Int J Equity Health. 2022 Sep 22;21:138. doi: 10.1186/s12939-022-01741-1 (PMC9493174; doi:10.1186/s12939-022-01741-1)
Supplement: Supplementary file 5 — Additional file 5: Table S4. Weight of each indicator from 2016 to 2020. [file 12939_2022_1741_MOESM5_ESM.docx]

**Additional file 5: Table S4.** Weight of each indicator from 2016 to 2020

| **Year** | **Public hospitals** | **Beds** | **Health technicians** | **Government financial subsidies** |
| --- | --- | --- | --- | --- |
| 2016 | 0.147 | 0.166 | 0.204 | 0.483 |
| 2017 | 0.138 | 0.161 | 0.198 | 0.504 |
| 2018 | 0.135 | 0.161 | 0.200 | 0.504 |
| 2019 | 0.135 | 0.165 | 0.210 | 0.490 |
| 2020 | 0.153 | 0.183 | 0.236 | 0.427 |
