# Supplementary material for: Equity and efficiency of public hospitals’ health resource allocation in Guangdong Province, China
Source: Int J Equity Health. 2022 Sep 22;21:138. doi: 10.1186/s12939-022-01741-1 (PMC9493174; doi:10.1186/s12939-022-01741-1)
Supplement: Supplementary file 6 — Additional file 6: Table S5. Comprehensive indicators of 21 cities in Guangdong Province from 2016 to 2020. [file 12939_2022_1741_MOESM6_ESM.docx]

**Additional file 6: Table S5.** Comprehensive indicators of 21 cities in Guangdong Province from 2016 to 2020

| **Cities (regions)** | **2016** | **2017** | **2018** | **2019** | **2020** |
| --- | --- | --- | --- | --- | --- |
| **PRD** | 89663.016 | 90751.842 | 95746.268 | 105595.176 | 121608.930 |
| GZ | 31268.076 | 31175.345 | 32580.372 | 35852.973 | 41326.861 |
| SZ | 15165.516 | 15924.134 | 17730.678 | 20522.296 | 24014.693 |
| ZH | 2945.660 | 3091.841 | 3332.393 | 3781.219 | 4530.180 |
| FS | 10539.750 | 10586.914 | 11184.786 | 12216.332 | 13801.741 |
| HZ | 5122.986 | 5270.993 | 5432.620 | 5842.593 | 6940.702 |
| DG | 8878.162 | 8865.333 | 9115.743 | 9695.947 | 11116.967 |
| ZS | 5162.102 | 5183.528 | 5309.548 | 5677.926 | 6061.566 |
| JM | 6025.743 | 6087.872 | 6255.188 | 6775.954 | 7832.273 |
| ZQ | 4555.022 | 4565.883 | 4804.940 | 5229.937 | 5983.948 |
| **ER** | 11855.387 | 12297.789 | 12839.548 | 13711.348 | 15785.792 |
| ST | 4800.785 | 5031.962 | 5276.418 | 5649.578 | 6500.134 |
| SW | 1617.816 | 1640.862 | 1715.899 | 1805.569 | 2011.764 |
| CZ | 1492.522 | 1487.578 | 1481.103 | 1594.556 | 1850.692 |
| JY | 3944.265 | 4137.387 | 4366.128 | 4661.646 | 5423.202 |
| **WR** | 16466.819 | 16918.805 | 17554.053 | 18766.566 | 21514.586 |
| YJ | 2985.193 | 3180.896 | 3279.189 | 3534.058 | 4058.667 |
| ZJ | 7501.059 | 7619.559 | 7832.684 | 8406.157 | 9725.419 |
| MM | 5980.566 | 6118.350 | 6442.180 | 6826.352 | 7730.500 |
| **MR** | 17624.747 | 17895.060 | 18743.030 | 20413.511 | 23237.257 |
| SG | 4730.303 | 4716.308 | 4948.510 | 5311.830 | 6001.384 |
| HY | 2527.701 | 2577.109 | 2851.093 | 3170.824 | 3667.173 |
| MZ | 4422.718 | 4490.996 | 4662.737 | 5035.656 | 5778.345 |
| QY | 3586.078 | 3634.513 | 3783.743 | 4195.743 | 4660.104 |
| YF | 2357.947 | 2476.133 | 2496.947 | 2699.459 | 3130.251 |

Note: PRD: Pearl River Delta; Eastern Region: ER; Western Region: WR; Mountainous Region: MR
