# Supplementary material for: Equity and efficiency of public hospitals’ health resource allocation in Guangdong Province, China
Source: Int J Equity Health. 2022 Sep 22;21:138. doi: 10.1186/s12939-022-01741-1 (PMC9493174; doi:10.1186/s12939-022-01741-1)
Supplement: Supplementary file 7 — Additional file 7: Table S6. The HRDI of the comprehensive indicators of 21 cities in Guangdong Province from 2016 to 2020. [file 12939_2022_1741_MOESM7_ESM.docx]

**Additional file 7: Table S6.** The HRDI of the comprehensive indicators of 21 cities in Guangdong Province from 2016 to 2020

| **Cities (regions)** | **2016** | **2017** | **2018** | **2019** | **2020** |
| --- | --- | --- | --- | --- | --- |
| **PRD** | 1.438 | 1.432 | 1.489 | 1.628 | 1.858 |
| GZ | 2.835 | 2.771 | 2.854 | 3.112 | 3.546 |
| SZ | 2.769 | 2.828 | 3.073 | 3.511 | 4.046 |
| ZH | 1.599 | 1.631 | 1.701 | 1.879 | 2.197 |
| FS | 1.829 | 1.811 | 1.886 | 2.041 | 2.296 |
| HZ | 0.641 | 0.654 | 0.667 | 0.710 | 0.837 |
| DG | 1.775 | 1.754 | 1.799 | 1.912 | 2.189 |
| ZS | 1.914 | 1.898 | 1.920 | 2.030 | 2.156 |
| JM | 0.909 | 0.916 | 0.935 | 1.008 | 1.159 |
| ZQ | 0.589 | 0.589 | 0.618 | 0.670 | 0.764 |
| **ER** | 0.740 | 0.770 | 0.805 | 0.861 | 0.993 |
| ST | 1.385 | 1.450 | 1.519 | 1.625 | 1.868 |
| SW | 0.442 | 0.451 | 0.473 | 0.499 | 0.558 |
| CZ | 0.522 | 0.521 | 0.519 | 0.559 | 0.651 |
| JY | 0.716 | 0.755 | 0.800 | 0.856 | 1.001 |
| **WR** | 0.733 | 0.752 | 0.777 | 0.829 | 0.948 |
| YJ | 0.664 | 0.705 | 0.725 | 0.778 | 0.891 |
| ZJ | 0.779 | 0.792 | 0.814 | 0.874 | 1.011 |
| MM | 0.726 | 0.740 | 0.774 | 0.816 | 0.920 |
| **MR** | 0.502 | 0.511 | 0.535 | 0.583 | 0.665 |
| SG | 0.654 | 0.652 | 0.683 | 0.733 | 0.828 |
| MZ | 0.553 | 0.564 | 0.589 | 0.639 | 0.737 |
| HY | 0.376 | 0.384 | 0.426 | 0.475 | 0.550 |
| QY | 0.417 | 0.421 | 0.437 | 0.484 | 0.536 |
| YF | 0.548 | 0.575 | 0.580 | 0.627 | 0.727 |

Note: PRD: Pearl River Delta; Eastern Region: ER; Western Region: WR; Mountainous Region: MR
